# Arrhythmogenic and antiarrhythmic actions of late sustained sodium current in the adult human heart

**DOI:** 10.1038/s41598-021-91528-1

**Published:** 2021-06-08

**Authors:** Anh Tuan Ton, William Nguyen, Katrina Sweat, Yannick Miron, Eduardo Hernandez, Tiara Wong, Valentyna Geft, Andrew Macias, Ana Espinoza, Ky Truong, Lana Rasoul, Alexa Stafford, Tamara Cotta, Christina Mai, Tim Indersmitten, Guy Page, Paul E. Miller, Andre Ghetti, Najah Abi-Gerges

**Affiliations:** AnaBios Corporation, 3030 Bunker Hill St., Suite 312, San Diego, CA 92109 USA

**Keywords:** Pharmacology, Cardiology

## Abstract

Late sodium current (late INa) inhibition has been proposed to suppress the incidence of arrhythmias generated by pathological states or induced by drugs. However, the role of late INa in the human heart is still poorly understood. We therefore investigated the role of this conductance in arrhythmias using adult primary cardiomyocytes and tissues from donor hearts. Potentiation of late INa with ATX-II (anemonia sulcata toxin II) and E-4031 (selective blocker of the hERG channel) slowed the kinetics of action potential repolarization, impaired Ca^2+^ homeostasis, increased contractility, and increased the manifestation of arrhythmia markers. These effects could be reversed by late INa inhibitors, ranolazine and GS-967. We also report that atrial tissues from donor hearts affected by atrial fibrillation exhibit arrhythmia markers in the absence of drug treatment and inhibition of late INa with GS-967 leads to a significant reduction in arrhythmic behaviour. These findings reveal a critical role for the late INa in cardiac arrhythmias and suggest that inhibition of this conductance could provide an effective therapeutic strategy. Finally, this study highlights the utility of human ex-vivo heart models for advancing cardiac translational sciences.

## Introduction

Development programs aiming to provide new treatments are frequently terminated prior to regulatory approval due to drug-induced cardiotoxicity or lack of adequate efficacy^1–5^. Clinical attrition is in part a consequence of the inability of current preclinical models, including artificially engineered cell lines, stem cell-derived platforms and animal models, to generate data predictive of human clinical responses^6,7^. The utilization of recently developed human ex vivo paradigms based on organ donor hearts provides novel opportunities for translational sciences^4,8^ and may be instrumental for informing the development of a new generation of therapies by identifying critical ion channel conductances underlying pathological states^9–15^.

At present, limited information is available on the role of late sodium current (INa) in normal as well as pathological states in the human heart. An increase in late INa function has been proposed to play a pivotal role in rhythm disorders^16,17^, heart failure^18–22^, and ischemia and hypoxia^23,24^. Consequently, a reduction of late INa could have therapeutic benefits, such as protection against drug-induced QT prolongation/pro-arrhythmia^25,26^, and may provide antiarrhythmic therapy for atrial fibrillation and ventricular tachycardia^27–31^.

Late INa contributes to the cardiac action potential morphology and is largely responsible for maintaining intracellular Na^+^ homeostasis^17,20, 22,31^. The current knowledge of the physiology and pathophysiology of late INa derives from studies conducted in rabbit, guinea pig, dog, and porcine hearts^32–43^, non-failing/failing human hearts or patients in sinus rhythm/atrial fibrillation^17,19, 44–48^. In the context of arrhythmia genesis, role for late INa has been well studied in animal models^17^. However, the specific contribution, if any, for late INa in the genesis or suppression of arrhythmias in human cardiomyocytes is still unknown. To address this knowledge gap, we performed a series of studies using adult human ex vivo preparations from normal and atrial fibrillation donor hearts to define the role of late INa in action potential morphology, intracellular Ca^2+^ handling, contractility, arrhythmogenesis, and the potential of late INa as a target for antiarrhythmic treatment and protection against drug-induced pro-arrhythmia.

## Results

### Detection and pharmacological modulation of late INa in human adult ventricular cardiomyocytes

**Figure 1:**
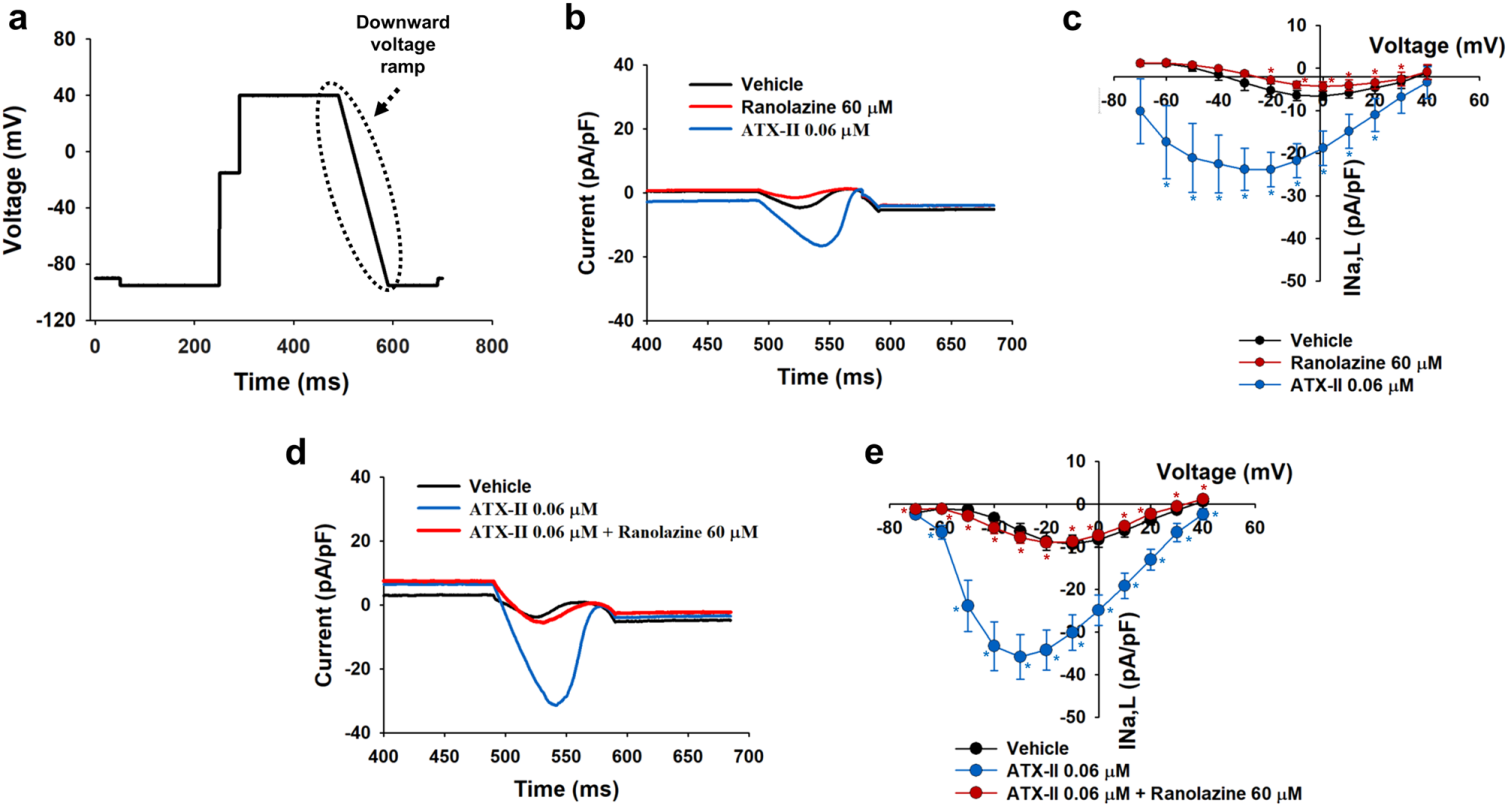
Characterization of late INa in adult human primary ventricular myocytes. (**a**) Schematic representation of the voltage ramp protocol to measure late INa. (**b**) and (**c**) Typical current recordings from an adult human primary ventricular myocyte and average current–voltage relationships of normal physiological late INa in the presence of Vehicle control and after exposure to either ranolazine (60 µM, n = 6 cells) or ATX-II (0.06 µM, n = 6 cells). (**d**) and (**e**) Effects of ranolazine on ATX-II-enhanced late INa. Representative recordings (**d**) and average current–voltage relationships (**e**) of late INa in the presence of Vehicle control and in the presence of ranolazine (60 µM) before and after application of ATX-II (0.06 µM, n = 6 cells). Data in (**c**) and (**e**) are expressed as mean ± SEM. Statistical analysis was conducted by ANOVA with a Bonferroni posthoc test. ^*,*^p < 0.05 versus vehicle control in (**c**) and (**e**), and ^*^p < 0.05 versus ATX-II in (**e**). Fitmaster analysis software package (HEKA Elektronik, Germany, www.heka.com) and SigmaPlot v14.0 (Systat Software Inc., CA, USA, www.systatsoftware.com) were used to generate the representative late INa current traces.

We first investigated the properties of late INa in human adult cardiomyocytes isolated from donor hearts. Utilizing voltage clamp electrophysiology in the whole cell configuration, we applied a voltage ramp protocol designed to mimic key features of a cardiac action potential waveform (Fig. 1a). The recordings show that the later part of the stimulus waveform, corresponding to the repolarization phase of the cardiac action potential, elicits a small inward current with peak amplitude at 0 mV (Fig. 1b, c). ATX-II toxin from Anemonia sulcata has been shown to be a selective enhancer of late INa^49^, whereas ranolazine, an anti-ischemic/antianginal drug, has been reported to be an inhibitor of late INa^50^. In our human adult cardiomyocyte system, ranolazine (60 µM) inhibited the inward current induced by the voltage ramp protocol (Fig. 1b, c). In contrast, ATX-II (0.06 µM) increased the inward current, an effect that was completely reversed by the addition of ranolazine (Fig. 1d, e). Furthermore, ATX-II caused a left shift in the current–voltage relationship of the inward current (Fig. 1c, e). These results are consistent with the inward current elicited by the stimulation protocol being largely carried by the late gating of the Nav1.5 channel and corresponding to late INa. These data provide evidence that isolated adult human cardiomyocytes express functional late INa.

### Contribution of late INa to the human ventricular action potential

**Figure 2:**
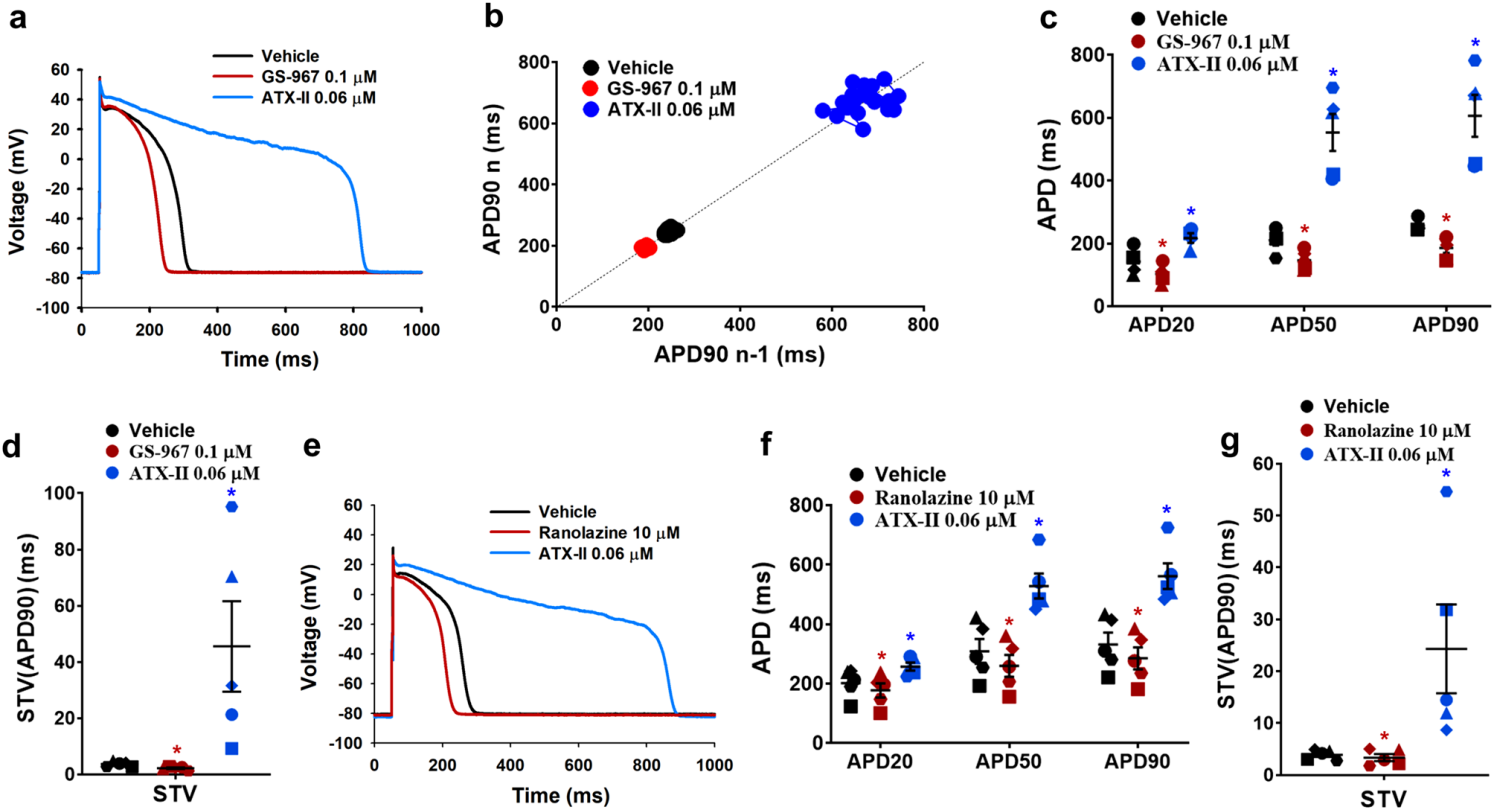
Physiological late INa and normal human ventricular action potential. (**a**) Typical action potentials (APs) recorded from a ventricular cell at a pacing of 1 Hz in the presence of vehicle control and after exposure to 0.1 µM GS-967 or 0.06 µM ATX-II. (**b**) shows a typical example of a Poincaré plot of APD90 from a ventricular cell (same cell as in **a**) under vehicle and in the presence of GS-967 or ATX-II. Note the large complex polygons during the application of ATX-II. (**c**) and (**d**) show mean % changes in APD (measured at 20% (APD20), 50% (APD50) and 90% (APD90) of repolarization) and STV(APD90), short-term variability of APD90 (n = 5 cells). (**e**) Typical APs recorded from a ventricular cell at a pacing frequency of 1 Hz in the presence of vehicle control and after exposure to 10 µM ranolazine or 0.06 µM ATX-II. (**f**) and (**g**) show mean % changes in APD and STV(APD90) under vehicle and in the presence of ranolazine or ATX-II (n = 5 cells). ^*,*^p < 0.05 versus values from vehicle control. Fitmaster analysis software package (HEKA Elektronik, Germany, www.heka.com) and SigmaPlot v14.0 (Systat Software Inc., CA, USA, www.systatsoftware.com) were used to generate the representative AP traces.

To investigate a possible role for late INa in cardiac action potential, we measured the effect of the selective late INa inhibitor GS-967^49,51^. In current clamp recording of action potentials induced by current injection in isolated adult human ventricular cardiomyocytes, we found that the application of 0.1 µM GS-967 decreased the action potential duration and reduced the temporal variability in action potential duration at 90% repolarization (APD90) between consecutive beats, a biomarker for pro-arrhythmia^52^, quantified as short-term variability in APD90 (STV(APD90)) (Fig. 2a–d). In contrast, ATX-II (0.06 µM) induced significant prolongation of the cardiomyocyte action potential and led to an increase of STV(APD90) (Fig. 2a–d). In another series of experiments, we used ranolazine to confirm the effects of late INa modulation on the cardiac action potential. Consistent with the effects observed with GS-967, the addition of 10 µM ranolazine shortened the action potential and decreased STV(APD90) (Fig. 2e–g). These data indicate that the late INa actively contributes to the cardiac action potential duration and kinetics such that suppression of this conductance results in shortening and reduced variability, whereas enhancement of the conductance leads to significant action potential prolongation and increased variability. The increase in short-term variability of action potential duration measured in the presence of ATX-II suggests that enhancement of late INa could have a pro-arrhythmic effect in human adult cardiomyocytes.

### Inhibition of late INa prevents ventricular repolarization abnormalities

**Figure 3:**
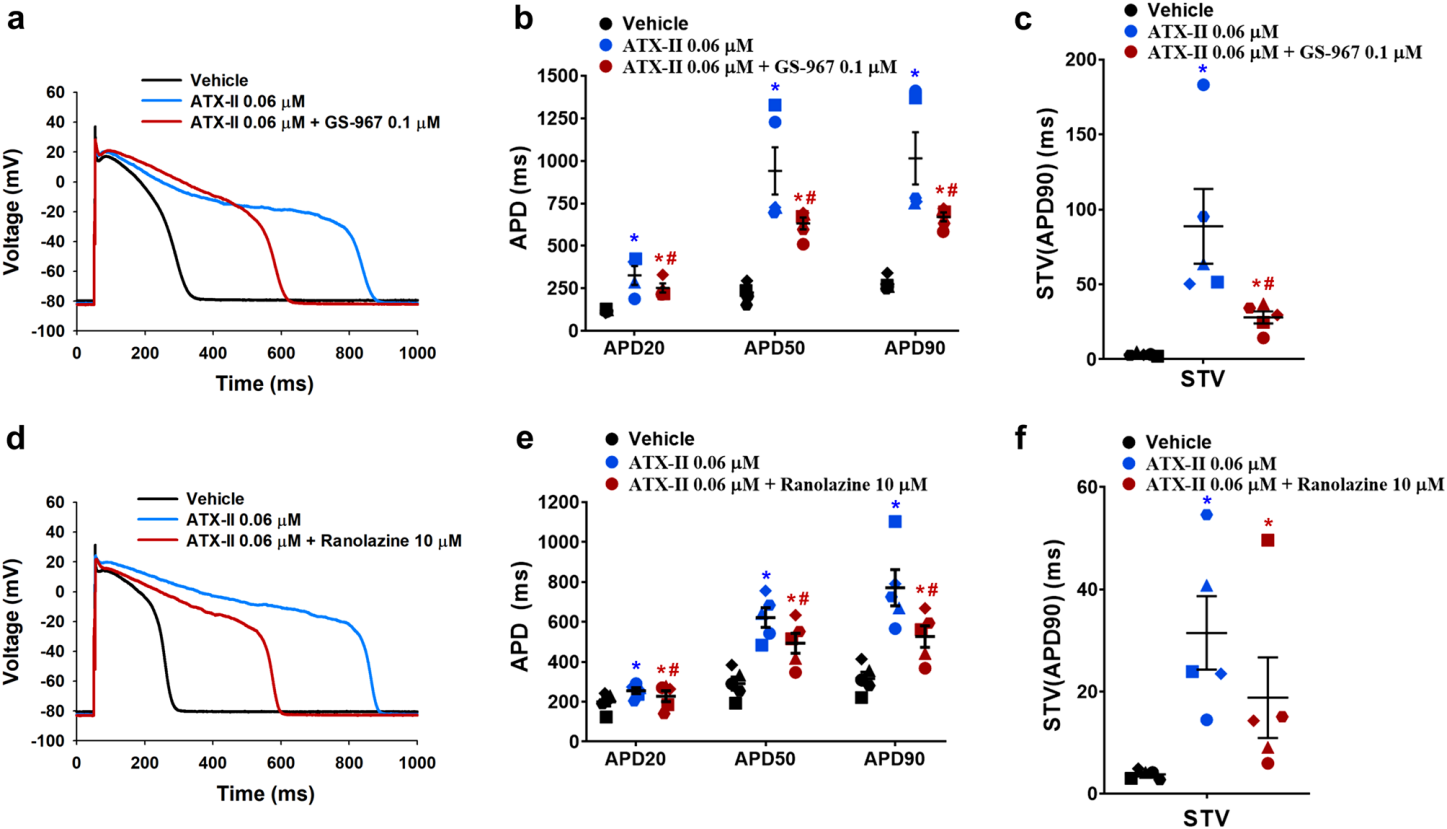
Effects of GS-967 and ranolazine on human ventricular action potential in condition mimicking LQTS3. (**a**) Typical action potentials (APs) recorded from a ventricular cell at a pacing of 1 Hz in the presence of vehicle control and after exposure to 0.06 µM ATX-II alone or in combination with 0.1 µM GS-967. (**b**) and (**c**) show mean % changes in APD (measured at 20% (APD20), 50% (APD50) and 90% (APD90) of repolarization) and STV(APD90), short-term variability of APD90 induced by addition of ATX-II alone or in the presence of GS-967 (n = 5 cells) at 1 Hz. (**d**) Typical APs recorded from a ventricular cell at a pacing frequency of 1 Hz in the presence of vehicle control and after exposure to 0.06 µM ATX-II alone or in combination with ranolazine 10 µM. (**e**) and (**f**) show mean % changes in APD and STV(APD90) induced by addition of ATX-II alone or in the presence of ranolazine (n = 5 cells) at 1 Hz. *,*p < 0.05 versus values from vehicle control. ^#^*p* < 0.05 versus values from ATX-II. Fitmaster analysis software package (HEKA Elektronik, Germany, www.heka.com) and SigmaPlot v14.0 (Systat Software Inc., CA, USA, www.systatsoftware.com) were used to generate the representative AP traces.

**Figure 4:**
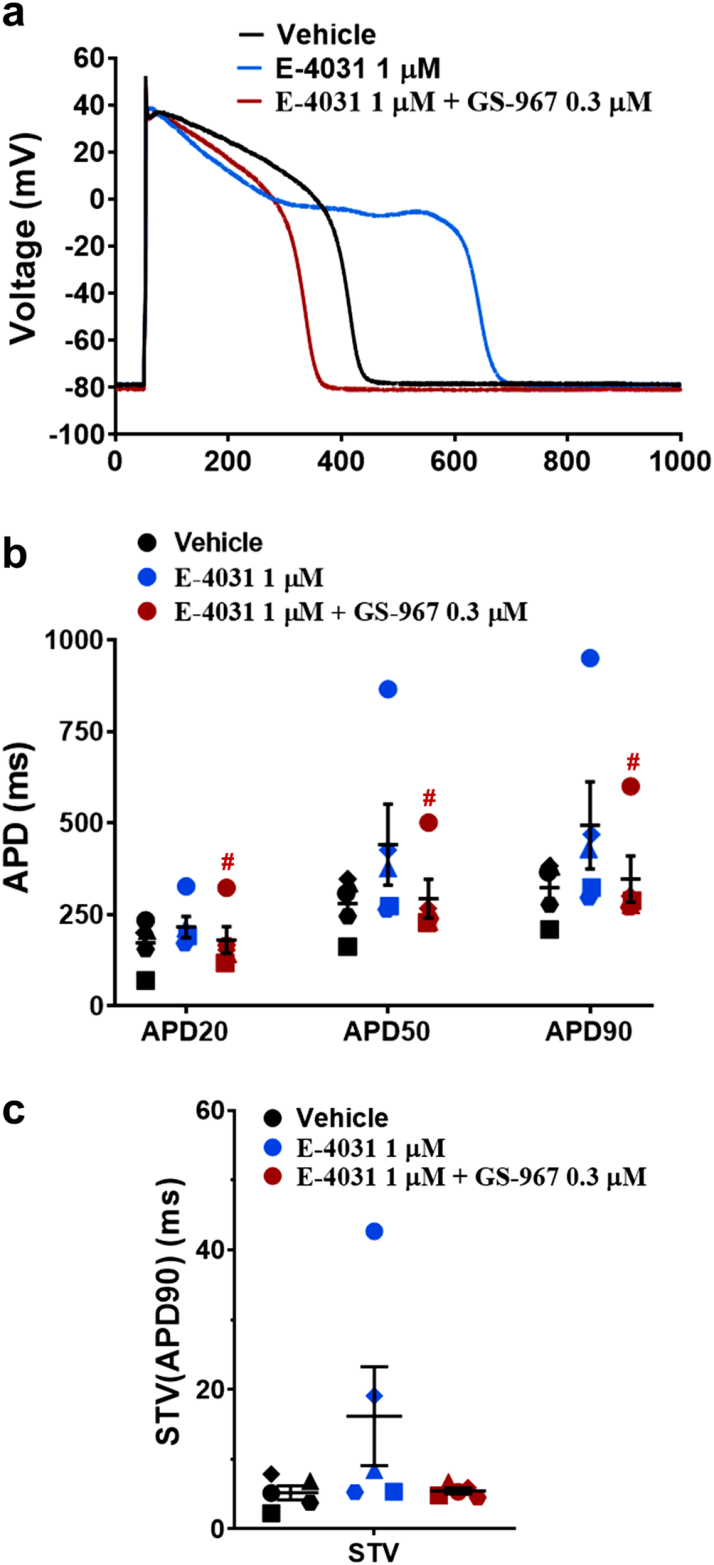
Effects of GS-967 on human ventricular action potential in condition mimicking LQTS2. (**a**) Typical action potentials (APs) recorded from a ventricular cell at a pacing of 1 Hz in the presence of vehicle control and after exposure to 1 µM E-4031 alone or in combination with 0.3 µM GS-967. (**b**) and (**c**) show mean % changes in APD (measured at 20% (APD20), 50% (APD50) and 90% (APD90) of repolarization) and STV(APD90), short-term variability of APD90 induced by addition of E-4031 alone or in the presence of GS-967 (n = 5 cells) at 1 Hz. ^#^*p* < 0.05 versus values from E-4031. Fitmaster analysis software package (HEKA Elektronik, Germany, www.heka.com) and SigmaPlot v14.0 (Systat Software Inc., CA, USA, www.systatsoftware.com) were used to generate the representative AP traces.

We next sought to determine the effects of late INa inhibition on ventricular repolarization abnormalities in adult human primary cardiomyocytes. In our experiments, repolarization abnormalities were induced by enhancement of late INa with ATX-II, a condition that mimics LQTS3, or by inhibition of IKr (rapidly activating delayed rectifier K^+^ channel)/hERG channel with the selective blocker E-4031^51^, a model for LQTS2. In one series of experiments, we determined the effects of GS-967 on repolarization abnormalities induced by the late INa agonist ATX-II at concentrations of 0.01 µM (Supplementary Fig. 1) and 0.06 µM (Fig. 3a–c). Perfusion of cardiomyocytes with ATX-II alone increased action potential duration and STV(APD90). The increase in repolarization variability was seen in all experiments carried out with ATX-II (Supplementary Fig. 1c; Fig. 3c). GS-967 at 0.3 µM (Supplementary Fig. 1) or 0.1 µM (Fig. 3a–c) reversed ATX-II-induced increases in action potential duration and re-stabilized the duration of repolarization. ATX-II-induced early afterdepolarization (EAD) events, another predictor of pro-arrhythmia^53^, were detected in 2 out of 9 cardiomyocytes (Supplementary Fig. 2a, b), and the addition of GS-967 suppressed the EAD events. We also found that ranolazine (10 µM) was effective in reverting ATX-induced action potential abnormalities and instability (Fig. 3d–f). Next, we assessed the effects of inhibition of late INa on repolarization abnormalities induced by inhibition of IKr. In isolated adult human cardiomyocytes, 1 µM E-4031 increased action potential duration and STV(APD90) (Fig. 4; Supplementary Fig. 2c). Addition of 0.3 µM GS-967 largely reversed E-4031-induced action potential prolongation (Fig. 4a, b) and restored action potential stability (Fig. 4c). EAD induced by E-4031 was also suppressed by the addition of 0.3 µM GS-967 (Supplementary Fig. 2c).

Considering the significant role that late INa appears to play in physiological as well as abnormal cardiac action potential, we investigated the extent of inter-donor variability of cardiomyocyte responses in the human population. A preliminary study of intra-heart variability of late INa contribution to action potential and the effects of ATX-II was conducted in 4 different donor hearts (Supplementary Fig. 3a). At 0.06 µM, ATX-II treatment resulted in significant ADP90 increase and no statistically significant difference was noted across cells from different hearts in the action potential-prolonging effects of ATX-II.

Taken together, these data demonstrate that late INa contributes to the human cardiac action potential kinetics and its potentiation can lead to repolarization abnormalities such as action potential prolongation, beat-to-beat variability, and EAD incidence while its inhibition can reduce the incidence of pro-arrhythmia markers.

### Inhibition of late INa reverses ventricular Ca^2+^ handling abnormalities

**Figure 5:**
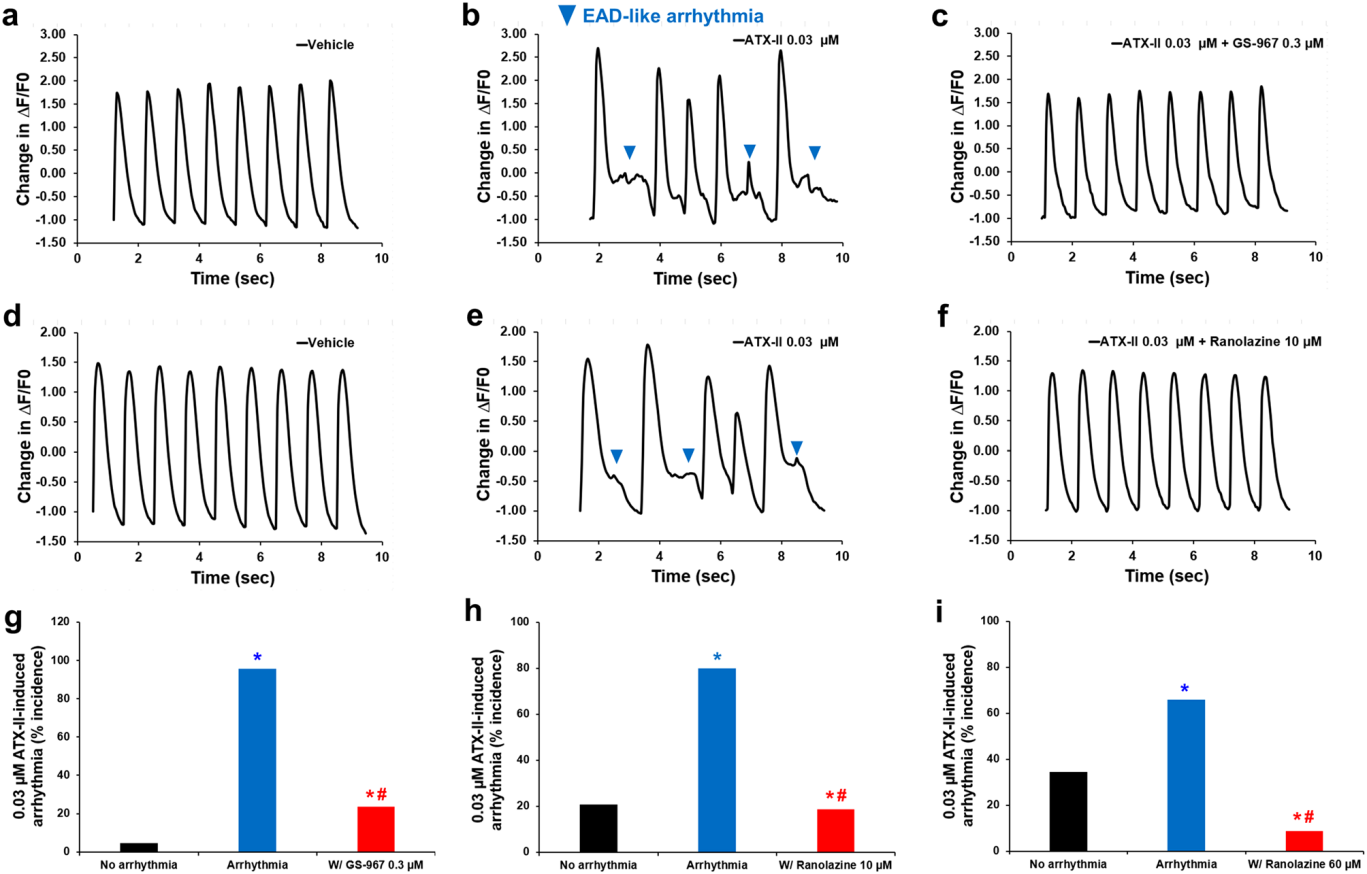
Effects of GS-967 and ranolazine on human ventricular Ca^2+^ transient in condition mimicking LQTS3. (**a**–**c**) and (**d**–**f**) show typical Ca^2+^ transients recorded from ventricular cells at a pacing of 1 Hz in the presence of vehicle control (**a** and **d**) and after exposure to 0.03 µM ATX-II alone (**b** and **e**) or in combination with 0.3 µM GS-967 (**c**) and 10 µM ranolazine (**f**). (**g**), (**h**) and (**i**) show mean % incidence in ATX-II-induced arrhythmia induced by addition of ATX-II alone or in the presence of GS-967 (**g**, n = 45 cells) or ranolazine (10 µM, **h**, n = 58 cells; 60 µM, **i**, n = 35 cells) at 1 Hz. *,*p < 0.05 versus values from vehicle control. ^#^*p* < 0.05 versus values from ATX-II. MetaMorph analysis software (Molecular Devices, CA, USA, www.moleculardevices.com) and a validated custom written MatLab program (The MathWorks Inc., MA, USA, www.mathworks.com) were used to generate the representative Ca^2+^ transients.

In cardiomyocytes, intracellular Ca^2+^ dynamics play a critical role in the contraction-excitation coupling and Ca^2+^ overload can lead to arrhythmias. To investigate the contribution of late INa to physiological and arrhythmic intracellular Ca^2+^ homeostasis in adult human primary ventricular cardiomyocytes, we assessed the impact of late INa potentiation or inhibition while measuring intracellular Ca^2+^ transients in isolated cardiomyocytes. We first evaluated the stability of Ca^2+^ dynamics in responses to 1 Hz pacing field stimulation and additions of vehicle solution. Neither the first or second vehicle addition affected Ca^2+^ transient (Supplementary Fig. 4a–d) and no statistically significant difference across cells from different donor hearts was observed (Supplementary Fig. 5). Addition of 0.3 µM GS-967 had no statistically significant effect on the Ca^2+^ transient (Supplementary Fig. 4e–g). In contrast, potentiation of late INa with ATX-II had profound effects on intracellular Ca^2+^ handling. ATX-II caused a profound delay in the decay of the Ca^2+^ transient signal and induced triggered EAD-like arrhythmic events (Fig. 5; Supplementary Fig. 6). GS-967 (0.3 µM, Fig. 5a–c, g) or ranolazine (10 µM (Fig. 5d–f, h) and 60 µM (Fig. 5i; Supplementary Fig. 6) restored normal Ca^2+^ dynamics and abolished ATX-II-induced arrhythmic events. Thus, the potentiation of late INa in conditions mimicking LQTS3 leads to abnormal and irregular Ca^2+^ dynamics which could underlie the arrhythmia markers observed in our action potential recordings, and inhibition of late INa restored physiological Ca^2+^ dynamics.

### Inhibition of late INa reverses abnormal ventricular and atrial contractions

**Figure 6:**
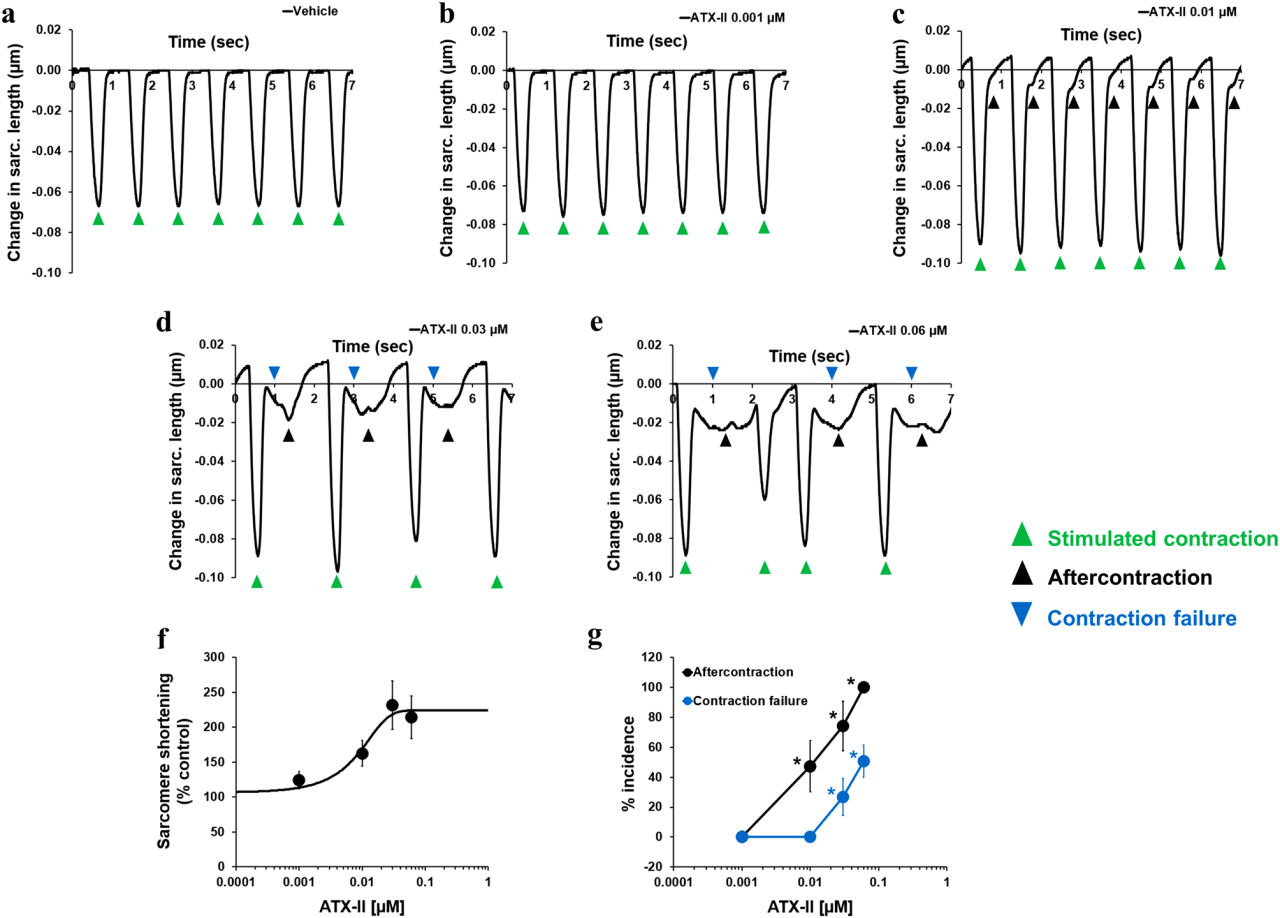
Effects of ATX-II on human ventricular contractility. (**a**–**e**) show typical contractility transients recorded from a ventricular cell at a pacing of 1 Hz in the presence of vehicle control and after exposure to four ascending concentrations of ATX-II. (**f**) shows ATX-effect curve for sarcomere shortening as a function of concentrations tested (EC_50_ = 0.012 µM, n = 6 cells). (**g**) shows mean % incidence of aftercontraction and contraction failure when cardiomyocytes were incubated with ATX-II at 1 Hz pacing frequency. *,*p < 0.05 versus values from vehicle control. IonWizard software (v1.2.22, IonOptix LLC, MA, USA, www.ionoptix.com) and SigmaPlot v14.0 (Systat Software Inc., CA, USA, www.systatsoftware.com) were used to generate the representative contractility transients and fitted C-E curve, respectively.

**Figure 7:**
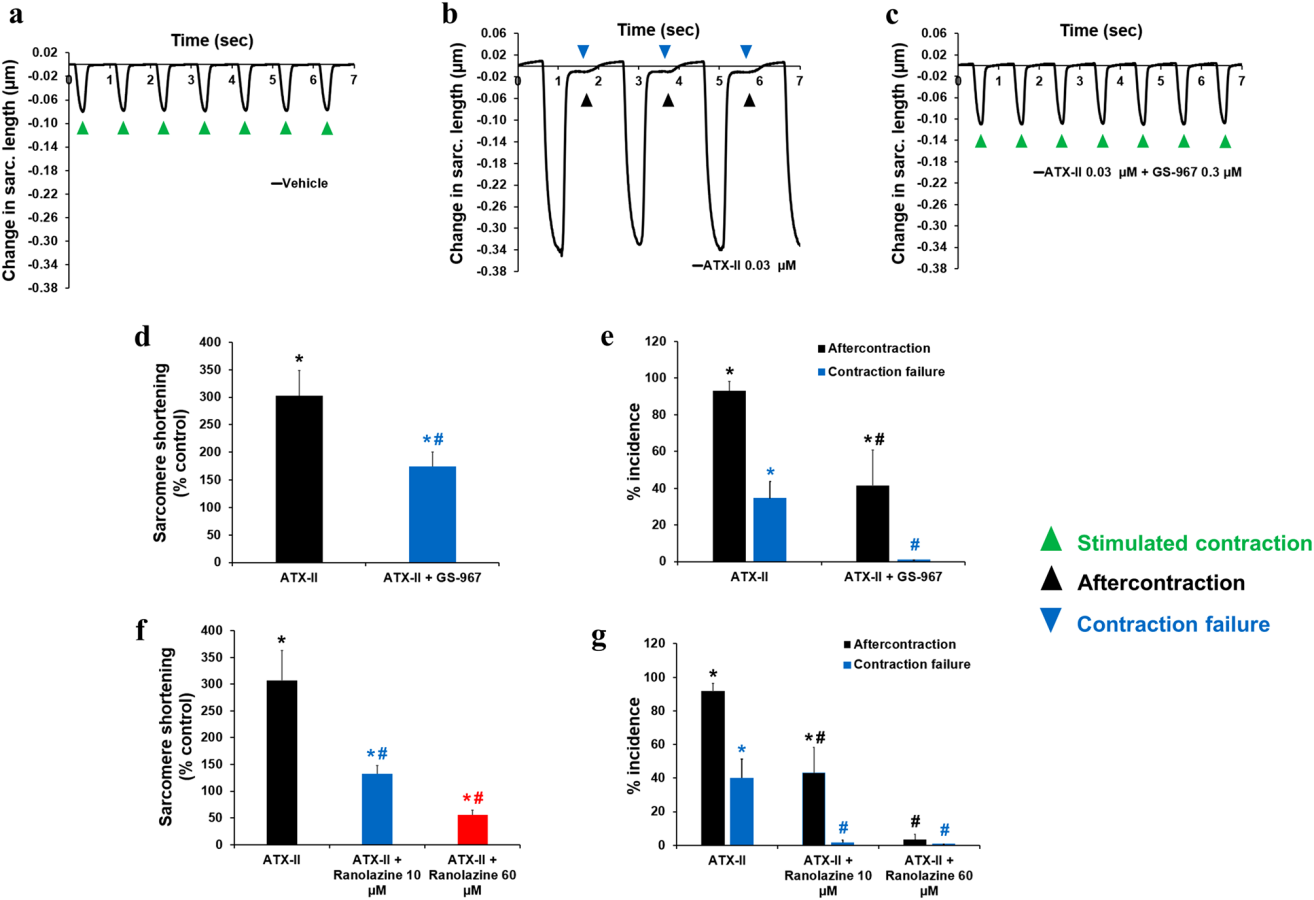
Effects of GS-967 and ranolazine on human ventricular contractility in condition mimicking LQTS3. (**a**–**c**) show typical contractility transients recorded from a ventricular cell at a pacing of 1 Hz in the presence of vehicle control (**a**) and after exposure to 0.03 µM ATX-II alone (**b**) or in combination with 0.3 µM GS-967 (**c**). (**d**) and (**e**) show mean % change in sarcomere shortening (**d**) and mean % incidence of aftercontraction and contraction failure (**e**) when cardiomyocytes were treated with 0.03 µM ATX alone or in combination with 0.3 µM GS-967 (n = 8 cells) at 1 Hz. (**f**) and (**g**) show mean % change in sarcomere shortening (**f**) and mean % incidence of aftercontraction and contraction failure (**g**) when cardiomyocytes were treated with 0.03 µM ATX alone or in combination with 10 and 60 µM ranolazine (n = 6 cells) at 1 Hz pacing frequency. *,*,*p < 0.05 versus values from vehicle control. ^#,#,#^*p* < 0.05 versus values from ATX-II. IonWizard software (v1.2.22, IonOptix LLC, MA, USA, www.ionoptix.com) was used to generate the representative contractility transients.

**Figure 8:**
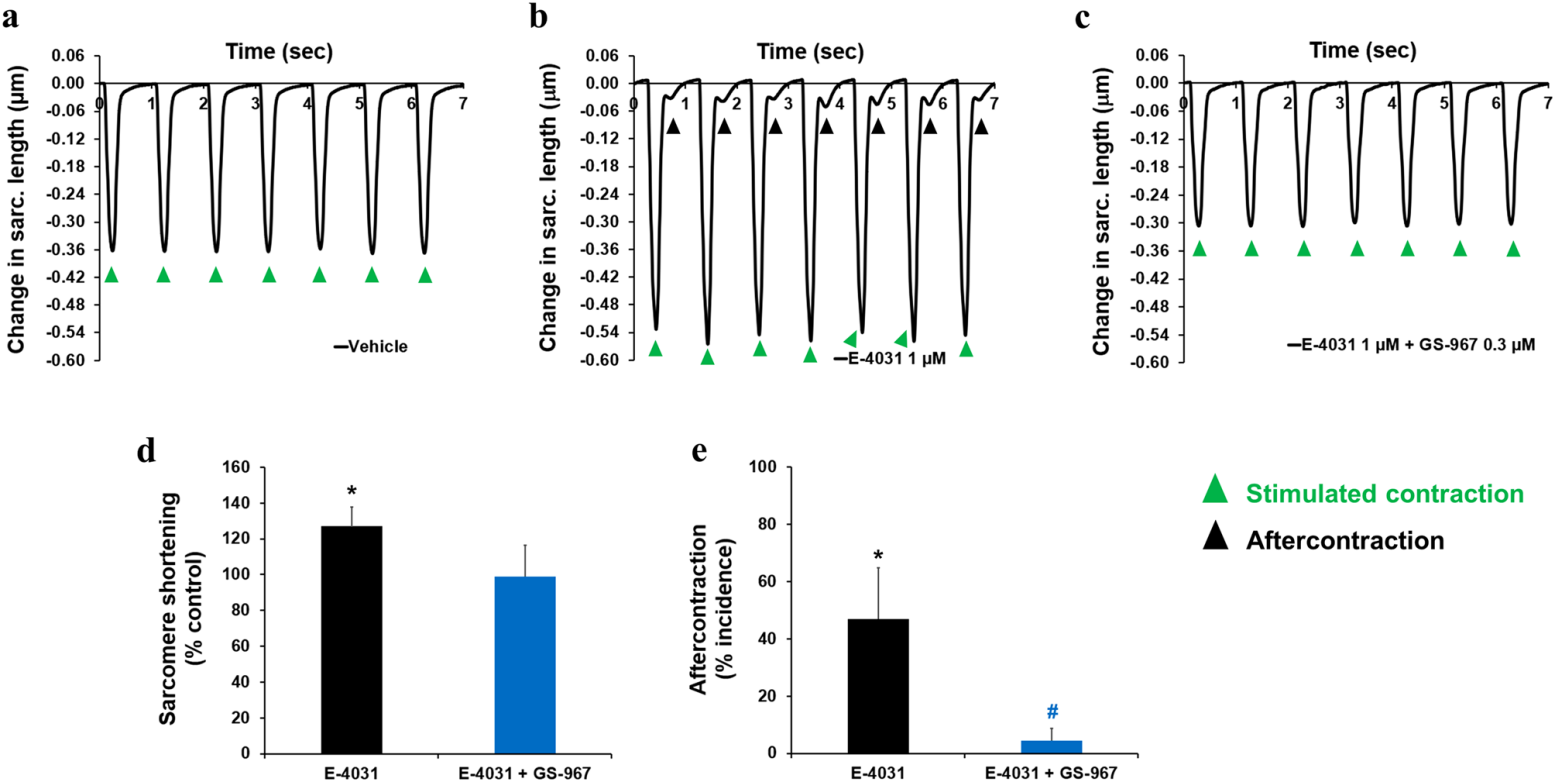
Effects of GS-967 on human ventricular contractility in condition mimicking LQTS2. (**a**–**c**) show typical contractility transients recorded from a ventricular cell at a pacing of 1 Hz in the presence of vehicle control (**a**) and after exposure to 1 µM E-4031 alone (**b**) or in combination with 0.3 µM GS-967 (**c**). (**d**) and (**e**) show mean % change in sarcomere shortening (**d**) and mean % incidence of aftercontraction and contraction failure (**e**) when cardiomyocytes were treated with 1 µM E-4031 alone or in combination with 0.3 µM GS-967 (n = 8 cells) at 1 Hz. *p < 0.05 versus values from vehicle control. ^#^*p* < 0.05 versus values from E-4031. IonWizard software (v1.2.22, IonOptix LLC, MA, USA, www.ionoptix.com) was used to generate the representative contractility transients.

Given the potent effects of late INa modulation on intracellular Ca^2+^ dynamics in adult human cardiomyocytes, we hypothesized that changes in late INa could impact cellular contractility. Consistent with this hypothesis, we found that ATX-II not only increased, in a concentration-dependent manner, the peak amplitude of sarcomere shortening, but also led to the manifestation of arrhythmic markers: i.e., aftercontraction and contraction failure (Fig. 6). We found that the positive inotropic effect of ATX-II was consistent across cells from 4 different donor hearts (Supplementary Fig. 3b). While enhancement of the late INa has a positive inotropic effect, we found that late INa inhibition by GS-967 (0.03–1 µM) had no effect on contractility (Supplementary Fig. 7a, b). This result enabled us to use GS-967 at 0.3 µM concentration to determine the contribution of late INa to ATX-II-induced abnormal ventricular contractility. Figure 7a–e shows that the addition of GS-967 at 0.3 µM restored normal contractility and significantly reduced the manifestation of arrhythmic markers. The role of late INa in ventricular contractility and arrhythmogenesis was also confirmed using ranolazine (Fig. 7f, g; Supplementary Fig. 7c–f). Next, we assessed the effects of inhibition of late INa on abnormal contractility caused by inhibition of IKr with E-4031. We found that 1 µM E-4031 caused a small increase in contractility (Fig. 8a, b, d) and led to the manifestation of aftercontraction (Fig. 8b, e), whereas the addition of 0.3 µM GS-967 prevented this increase in contractility (Fig. 8c, d) and restored the stability of contractions (Fig. 8c, e). These data suggest that aftercontraction and contraction failure are the two main markers of ventricular arrhythmogenesis in condition mimicking LQTS3, whereas aftercontraction is the principal marker of arrhythmia in condition mimicking LQTS2.

**Figure 9:**
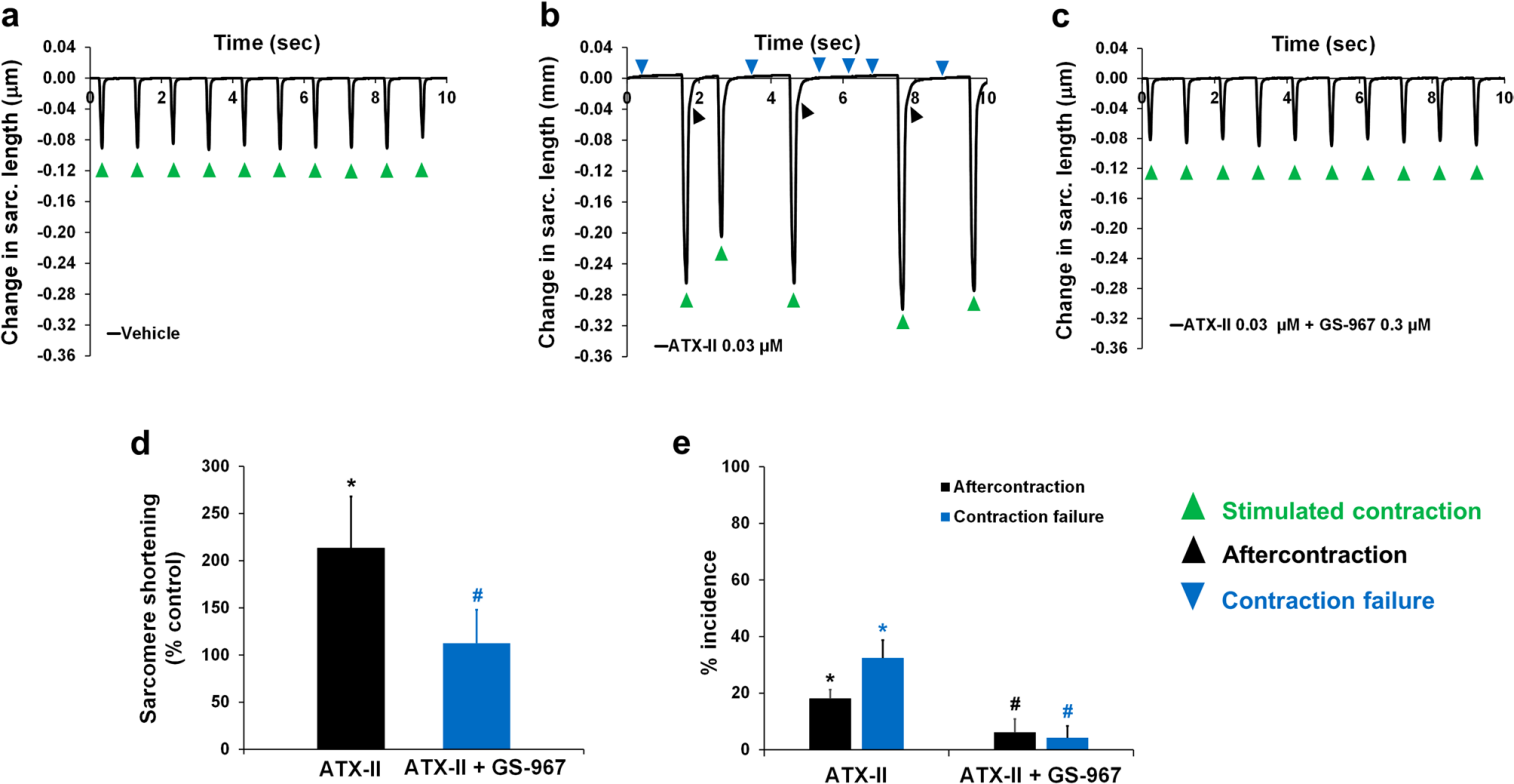
Effects of GS-967 on human atrial contractility in condition mimicking LQTS3. (**a**–**c**) show typical contractility transients recorded from a ventricular cell at a pacing of 1 Hz in the presence of vehicle control (**a**) and after exposure to 0.03 µM ATX-II alone (**b**) or in combination with 0.3 µM GS-967 (**c**). (**d**) and (**e**) show mean % change in sarcomere shortening (**d**) and mean % incidence of aftercontraction and contraction failure (**e**) when cardiomyocytes were treated with 0.03 µM ATX-II alone or in combination with 0.3 µM GS-967 (n = 5 cells) at 1 Hz. *,*p < 0.05 versus values from vehicle control. ^#,#^*p* < 0.05 versus values from ATX-II. IonWizard software (v1.2.22, IonOptix LLC, MA, USA, www.ionoptix.com) was used to generate the representative contractility transients.

The results we obtained from human adult ventricular cardiomyocytes prompted us to test whether the late INa conductance plays a similar role in atrial cardiomyocytes. We observed that ATX-II can induce positive inotropy and arrhythmia markers in adult human atrial cardiomyocytes and GS-967 restored normal contractions (Fig. 9a–d). Moreover, ATX-II reduced the incidence of aftercontraction and contraction failure (Fig. 9b, c, e).

Isolated adult human cardiomyocytes provide an extremely convenient preparation for mechanistic, biophysical, and pharmacological studies. However, to rule out the possibility that some of our findings may be attributed to abnormalities in the physiology of isolated cells, we evaluated the arrhythmogenic role of late INa in multicellular atrial and ventricular tissue preparations from normal donor hearts. In both cardiac tissue types, ATX-II induced positive inotropy (Supplementary Fig. 8a, b, d, e, g) and arrhythmia markers (Supplementary Fig. 8b, e, h), whereas 0.3 µM GS-067 reversed these effects (Supplementary Fig. 8c, f–h). In addition, late INa inhibition in atrial tissues prevented ATX-II-induced development of enhanced automaticity, a marker of atrial ectopic activity (Supplementary Fig. 8b, c)^54^.

### Inhibition of late INa reverses arrhythmia in donors with atrial fibrillation

**Figure 10:**
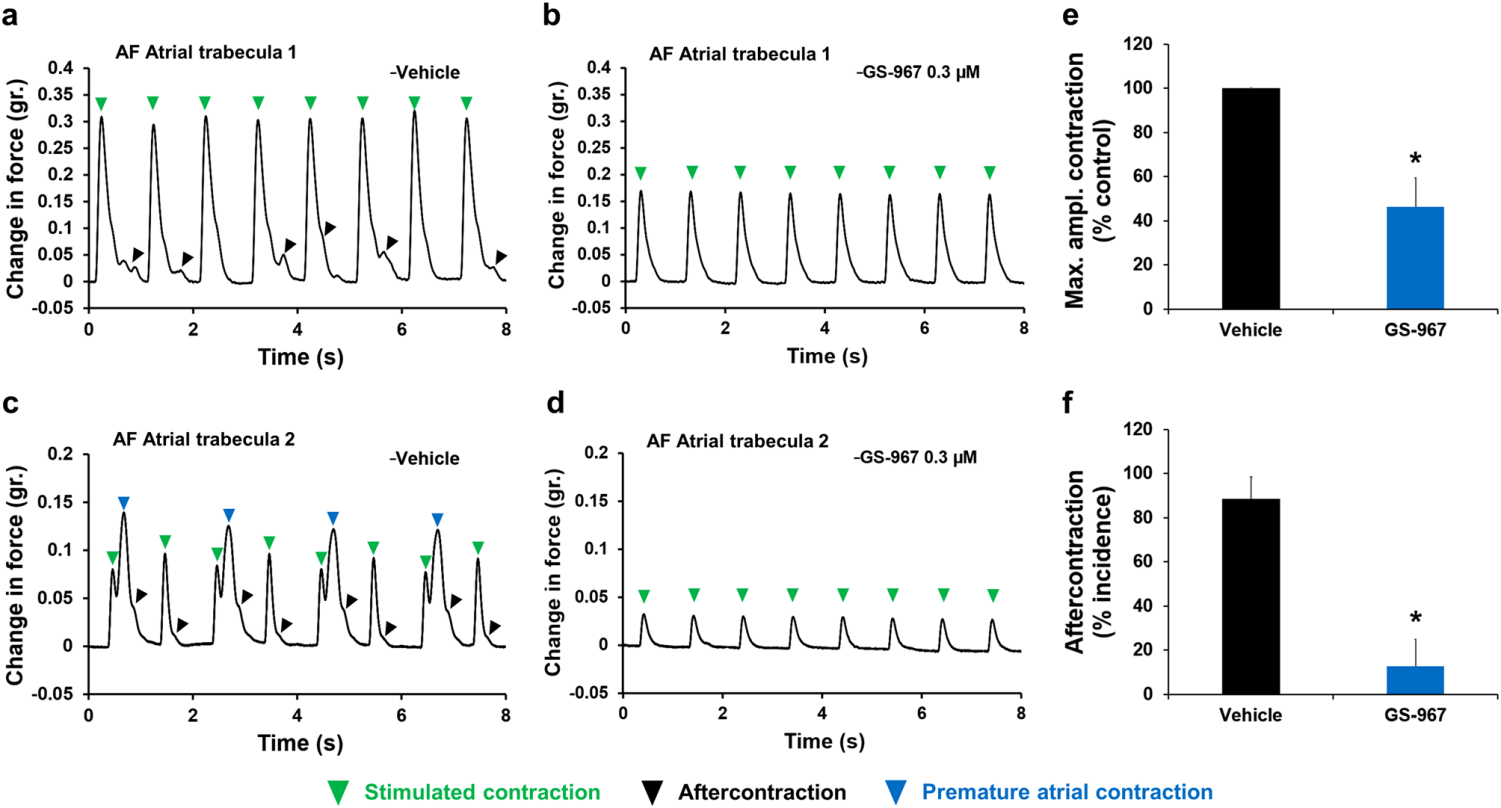
Effects of GS-967 on human atrial trabeculae contraction from donors with atrial fibrillation (AF) in condition of spontaneous arrhythmia. (**a**–**b**) and (**c**–**d**) show typical contraction/relaxation cycles recorded from two atrial trabecula of donors with AF at a pacing of 1 Hz in the presence of vehicle control (**a** and **c** with incidence of spontaneous arrhythmic markers: aftercontraction (**a** and **c**) and premature atrial contraction (**c**)) and after exposure to 0.3 µM GS-967 (**b** and **d** with no incidence of arrhythmic markers). (**e**–**f**) show mean % control in maximum amplitude of contraction (Max. ampl. Contraction) (**e**) and mean % incidence of aftercontraction (**f**) when atrial trabeculae were treated with vehicle or after exposure to 0.3 µM GS-967 (n = 4 trabeculae) at 1 Hz. *p < 0.05 versus values from vehicle. LabChart Software (v8.1.16, ADInstruments Inc., CO, USA, www.adinstruments.com) was used to generate the representative contraction/relaxation cycles.

Late INa inhibiting drugs have been proposed as potential antiarrhythmic agents for treatment of atrial fibrillation^17^. To investigate the contribution of late INa to atrial fibrillation, we assessed the contribution of late INa to manifestation of arrhythmic markers in contraction transients measured from atrial trabeculae obtained from donors with atrial fibrillation. We found that in donors with atrial fibrillation, atrial trabeculae exhibited spontaneous arrhythmic markers, aftercontraction (Fig. 10a, c), and premature atrial contraction (a marker and predictor of atrial ectopy and atrial fibrillation, respectively; Fig. 10c)^55^. This finding demonstrates the occurrence of atrial arrhythmogenic remodeling. Addition of 0.3 µM GS-967 restored normal atrial contractions (Fig. 10b, d), reduced amplitude of contraction (Fig. 10b, d, e), and prevented atrial remodeling-induced development of aftercontractions and premature atrial contractions (Fig. 10b, d, f). However, ventricular trabeculae from the same donors did not exhibit abnormalities in contractility or spontaneous arrhythmia markers. These data suggest that late INa is central to atrial arrhythmogenic remodelling and promotion of atrial fibrillation. It is important to note that spontaneous arrhythmic markers were not seen in all atrial tissues tested from hearts of donors with atrial fibrillation. However, sub-maximal concentration of ATX-II led to aftercontractions in atrial tissues from atrial fibrillation donors (Supplementary Fig. 9a, c) and addition of GS-967 at 0.3 µM blocked the occurrence of arrhythmias (Supplementary Fig. 9b, c). Taken together, these data suggest that late INa inhibitors can be useful in treating atrial fibrillation.

## Discussion

We have investigated the functional expression and the pathophysiological role of late INa in both ventricular and atrial cells and tissue preparations from normal and atrial fibrillation donor hearts. The main findings of the present investigation can be summarized as follows: (1) adult human ventricular cardiomyocytes express functional late INa; (2) late INa contributes to the normal human ventricular action potential plateau conductances; (3) enhancement of late INa can mimic LQTS3 and LQTS2 conditions and result in prolongation of ventricular action potential, with concomitant increase in the instability of action potential duration; (4) enhanced late INa generates abnormal ventricular Ca^2+^ dynamics; (5) treatments that enhance late INa and inhibit IKr increase ventricular and atrial contractility and cause arrhythmic contractions; (6) atrial tissues from donor hearts affected by atrial fibrillation exhibit spontaneous arrhythmic markers in the absence of drug treatment and inhibition of late INa led to a significant reduction of arrhythmia markers, indicating a possible direct contribution of late INa to atrial fibrillation and a therapeutic approach targeting this conductance.

The voltage protocol we used to record late INa was selected to better model the action potential plateau phase and its influence on phase 3 repolarization, compared to a classic square voltage step. This protocol was recently adopted by the CiPA (Comprehensive In Vitro Pro-arrhythmia Assay) initiative^56,57^. The potencies of late INa modulators, including ATX-II and ranolazine, were recently reported using this protocol^56,57^. We relied on ranolazine/GS-967 and ATX-II as gating modifiers to inhibit and enhance late INa, respectively. We found that, compared to cel lines stably expressing human Nav1.5 channels^56, 57^ and rabbit ventricular myocytes^51^, human primary cardiomyocytes and trabeculae are 2.5- to fivefold more sensitive to these gating modilators. Consequently, for drugs acting on late INa, extrapolation of potency values from artificial cellular abd animal models to human, will require particular caution^4^. Additionally, ATX-II-augmented late INa inward current observed in our study likely reflects the reactivation of channels that recovered from inactivation^57^. This mechanism appears to differ from the enhancement of late INa that is effected by veratridine, which leads to the stabilization of the open conformation of the channel^56,57^. Therefore, late INa translational research could certainly benefit from a detailed characterization of the arrhythmogenic action of veratridine in human cardiomyocytes and tissues. Previous work on late INa in human cardiomyocytes utilized cells isolated from normal donor hearts and failing explanted hearts; these studies reported a small current density of 0.34 pA/pF for late INa^44^. Similarly, recent studies employing artificially engineered cell lines^56,57,58,59^ and cardiomyocytes from animals or hiPSC-derived cardiomyocytes also reported a small late INa density (< 1 pA/pF)^49,51,60–64^. In contrast, we have observed a late INa density in a range of 6.5 to 9.3 pA/pF. The cause for the discrepancy is unclear but it could originate from the specific recording conditions, the different stimulus waveforms used in the studies, distinct expression of Na^+^ channels, different molecular structure of Na^+^ channels, or cross-species difference of late INa.

We found that under physiological conditions, late INa contributes to the ventricular action potential in the adult human heart and inhibition of this conductance results in action potential shortening and decreases the beat-to-beat variability of repolarization. These findings are consistent with previous studies reporting that tetrodotoxin (TTX), an inhibitor of late INa, shortened the action potential in human cardiomyocytes from normal and failing donor hearts^43, 44^ and reduced beat-to-beat variability of repolarization in cells from failing donor hearts^44^. Our data also highlights a potential cross-species difference in the contribution of late INa to the cardiac action potential, whereby cardiomyocytes from guinea pig, rabbit, and dog hearts exhibit a significantly smaller late INa conductance compared to human cardiomyocytes^43,50,51,61,63,65^. Moreover, it is well established that aberrant cardiomyocyte excitation–contraction coupling can lead to irregular heartbeat and contractile dysfunction^66^. We demonstrate that under LQTS3- and LQTS2-mimicking conditions, enhanced late INa causes repolarization abnormalities, dysregulation of Ca^2+^ handling and pro-arrhythmia, and inhibition of late INa can reverse these effects. Most importantly, our findings are also consistent with the results of clinical studies that evaluated the effects of late INa blockers, like ranolazine, mexiletine and lidocaine, and found that late INa inhibition offers protection against abnormal ventricular repolarization to normal subjects treated with an IKr/hERG channel blocker (Dofetilide)^25,26,67^ or patients with sustained late INa due to LQTS2 and LQTS3^68,69^. Moreover, this study is the first to confirm that mechanisms of human action potential arrhythmogenesis in LQTS3 and LQTS2 conditions consist of action potential prolongation and increase in dispersion of repolarization and EAD incidence. Potential arrhythmogenesis in LQTS3 and LQTS2 conditions related to their mechanisms of action have also been reported in cardiomyocytes from different animal species. Our action potential data agree with studies performed with rabbit, guinea pig, dog, and porcine cardiomyocytes^49,51,63,64,70–72^, as well as dog Purkinje fibers^50^. These studies reported that inhibition of late INa prevents and reverses pro-arrhythmic effects (i.e., action potential prolongation, action potential duration variability, and EAD) of ATX-II and three IKr/hERG inhibitors E-4031, dofetilide and sotalol. Furthermore, cardiomyocytes from failing human hearts were reported to exhibit repolarization abnormalities, action potential prolongation and increase in dispersion of repolarization and EAD incidence^44^. Suppression of these abnormalities with TTX is indicative of late INa enhancement as a causal factor in the remodeling process and arrhythmia in failing hearts. It is therefore important in future studies to comprehensively determine human action potential arrhythmogenic mechanisms in different heart disease conditions, such as heart failure.

Our investigation provides first evidence that, in human adult cardiomyocytes, enhancement of late INa leads to dysregulated Ca^2+^ handling (i.e., EAD-like events and triggered transients) and causes arrhythmic contractions (i.e., aftercontraction and contraction failure events). Consequently, arrhythmias induced by late INa potentiation could result from large Ca^2+^ entry (through prolonged and dispersed action potential) and abnormal diastolic Ca^2+^. We propose the following mechanistic sequence to explain the anomalous diastolic Ca^2+^: (1) enhanced late INa leads to excessive Na^+^ loading, (2) increase in intracellular Na^+^ inhibits the forward-mode of the Na^+^/Ca^2+^ exchanger and eventually the exchanger, via its reverse-mode, causing an influx of Ca^2+^, and (3) influx of Ca^2+^ causes spontaneous Ca^2+^ release from the sarcoplasmic reticulum. Thus, anomalous diastolic Ca^2+^ dynamics could act as the trigger for arrhythmias, while increased dispersion of action potential repolarization could provide the substrate. Additionally, our data indicate that LQTS3-mimicking condition (such as during the perfusion of ATX-II) led to a higher incidence of late INa-mediated abnormal ventricular repolarization and contractile function compared to LQTS2 condition, as observed during perfusion with E-4031. These data suggest that the LQTS3 condition is associated with a high prevalence of the substrate and trigger compared to the LQTS2 condition, possibly indicating a difference in severity of arrhythmias between the two conditions. This finding agrees with clinical studies reporting higher lethality of arrhythmia in patients with LQTS3 compared to LQTS2^73–75^. Additionally, we also observed that inhibition of ATX-II-enhanced late INa likewise reduced arrhythmic markers and restored normal rhythm in human atrial myocytes. This result suggests that late INa-induced impaired atrial Na^+^ homeostasis and increased diastolic Ca^2+^ may also play a key role in atrial arrhythmias. Similarly, enhanced late INa induced arrhythmias in human atrial and ventricular trabeculae. In both types of tissue, late INa inhibitors restored normal contractility. While manifestation of arrhythmic markers was limited to aftercontraction in ventricular trabeculae, enhanced automaticity and aftercontraction were observed in atrial trabeculae. The data from tissue preparations (trabeculae) also suggest that late INa-induced arrhythmia markers were not artificially generated by the lack of cell-to-cell coupling in isolated human cardiomyocyte preparations. Furthermore, our findings with atrial cardiomyocytes and trabeculae agree with studies reporting the presence of late INa in the atria of patients in sinus rhythm and atrial fibrillation^48^ and its contribution to atrial arrhythmias^47^. We also report that responses to late INa modulators, ATX-II and ranolazine, were similar in the human atria and ventricle, although a greater sensitivity to these two modulators was recorded in rabbit atrial than ventriculae cardiomyocytes^40^. This finding is yet another example that data from animal hearts should be interpreted with caution when extrapolating to humans.

Late INa inhibitor, ranolazine, is known to terminate premature atrial contractions, which is a clinical marker and predictor of atrial ectopy and atrial fibrillation, respectively^55^, in human atrial trabeculae from patients in sinus rhythm exposed to high extracellular Ca^2+^ or Isoprenaline^47^. In addition, the current investigation provides the first evidence for the manifestation of spontaneous arrhythmic markers, including aftercontraction and premature atrial contraction, in atrial trabeculae from organ donors with atrial fibrillation. This spontaneous arrhythmia is possibly the result of atrial electrical remodeling and its inhibition with late INa inhibitor GS-967 may be a feature of a sub-population of patients with atrial fibrillation who may experience therapeutic benefit from the pharmacological inhibition of late INa current. Moreover, we demonstrated that inhibition of late INa causes a significant reduction in spontaneous arrhythmic behavior in tissue from donors affected by atrial fibrillation. This finding agrees with recent clinical observations reporting that ranolazine decreases incidence of atrial fibrillation in various clinical settings, such as after cardiac surgery, in acute coronary syndromes and post-electrical cardioversion^29^ and reduces its recurrence in patients with the persistent type^28^. The data we obtained using atrial trabeculae isolated from donors with atrial fibrillation also agree with clinical reports of lower incidences of atrial fibrillation and ventricular tachyarrhythmia in patients with acute coronary syndrome treated with late INa inhibitors^76^. In our study too, we observed that not all atrial trabeculae from atrial fibrillation donors exhibit spontaneous contractions and arrhythmia markers. Therefore, future studies will be needed to address the heterogeneity of atrial electrical remodeling in the atrial fibrillation patient population, which will further clarify the potential therapeutic value of late INa inhibition. Given the cross-species differences we have discussed between human and animal cardiomyocytes with regards to the properties and amplitude of the late INa conductance, therapeutic development efforts that are exclusively reliant on animal models run the risk of selecting molecules with limited utility for the treatment of human atrial fibrillation^77–80^. The availability of human primary cardiac tissue and cells for healthy and atrial fibrillation donors provides a unique opportunity to identify and optimize novel molecules with the activity profile needed for effective treatment of specific atrial fibrillation patient populations^4,8–15,81,82^.

In conclusion, the present study provides extensive insight into the arrhythmogenic and antiarrhythmic actions of late INa in the human heart. In addition, the human paradigm of isolated cardiac tissues and cells that we have presented could be instrumental in uncovering novel drugs aimed at various forms of heart arrhythmia and disease.

## Methods

### Donor heart procurement

All methods were carried out in accordance with relevant guidelines and regulations. All human hearts used for this study were non-transplantable and ethically obtained by informed legal consent (first person or next-of-kin) from cadaveric organ donors in the United States (US). Our recovery protocols and in vitro experimentation were pre-approved by IRBs (Institutional Review Boards) at transplant centers within the US OPTN (Organ Procurement Transplant Network). Furthermore, all transfers of the donor hearts are fully traceable and periodically reviewed by US Federal authorities. Donor characteristics, heart number, and donor identifier are shown in Supplementary Table 1 and exclusion criteria were previously described^9^.

### Cardiomyocyte electrophysiology

Upon arrival at our laboratory, hearts were re-perfused with ice-cold proprietary cardioplegic solution and adult human primary ventricular myocytes were isolated enzymatically from the ventricles^11, 14^.

The whole-cell configuration of the patch-clamp technique was used to record late INa. The cells were held in the voltage-clamp mode using an EPC-10 USB amplifier (HEKA Elektronik, Germany). The protocol, shown in Fig. 1a, was elicited every 10 s. Cells were held at a potential of − 80 mV for 50 ms. Following a hyperpolarizing pulse to − 95 mV for 200 ms, pulses to − 20 mV and 40 mV were then applied for 50 and 200 ms to fully activate the peak INa current and inactivate the ICa current, respectively. For measuring drug effects on the current–voltage relationship of late INa, a downward voltage ramp of 100 ms from 40 to − 95 mV was applied (Fig. 1a). Series resistance were compensated at 40% and current measurements were normalized to cell capacitance to estimate late INa density. Patch pipettes were pulled from filamented borosilicate glass (Warner Instruments LLC, CT, USA) using a P-97 puller (Sutter Instruments, CA, USA) and had tip resistances of approx. 4 MΩ when filled with the internal solution. All recordings were carried at approx. 35 °C and signals were acquired at a sampling rate of 10 kHz, filtered at 1 kHz and analyzed offline using the Fitmaster analysis software package (HEKA Elektronik, Germany). The composition of the internal solution was as follows (in mM): 130 CsCl, 7 NaCl, 5 MgATP, 5 EGTA, 5 HEPES and 1 MgCl_2_–H_2_O; pH adjusted to 7.2 with CsOH and osmolarity to 275 mOsm. The composition of the external solution was as follows (in mM): 130 NaCl, 10 HEPES, 10 Glucose, 4 CsCl, 2 CaCl_2_ and 1 MgCl_2_; pH adjusted to 7.4 using NaOH and osmolarity to 295 mOsm.

For current-clamp action potential (AP) recordings, the internal solution contained (in mM): 100 L-Aspartic Acid K^+^ Salt, 25 KCl, 10 EGTA, 5K_2_-ATP, 5 HEPES and 1 MgCl_2_–6H_2_O; pH adjusted at 7.2 using KOH and osmolarity to 275 mOsm. The composition of the external solution was as follows (in mM): 140 NaCl, 5.4 KCl, 5.5 Glucose, 5 HEPES, 1.8 CaCl_2_, 1 MgCl_2_ and 0.33 Na_2_HPO_4_; pH adjusted to 7.4 using NaOH and osmolarity to 295 mOsm. APs were recorded at approx. 35 °C with pipettes which were pulled from filamented borosilicate glass and had tip resistances of approx. 4 MΩ when filled with the internal solution. The pipette was connected to the headstage of an EPC-10 USB amplifier (HEKA Elektronik, Germany). AP signals were acquired using PatchMaster software (HEKA Elektronik, Germany). Myocytes were first stimulated with a current pulse of 3 ms duration with increments of 50 pA to determine the rheobase that evokes an AP. Next, AP recordings were elicited using a stimulation of 50% above the current pulse threshold and repeated every second (i.e., 1 Hz pacing frequency). Additional offline analysis, using a validated custom written MatLab program (The MathWorks, Inc., MA, USA), was carried out and AP duration (APD) was measured at 20%, 50% and 90% repolarization (APD20, APD50 and APD90, respectively). Data for each experimental condition were the mean of 20 APDs. Beat-to-beat variability of repolarization was quantified as short-term variability of APD90 (STV(APD(90)) from Poincare plots over a period of 20 s, which was calculated as ∑|*APD*_*n*+1_ − *APD*_*n*_|/[*n*_beats_ × √2]^9^. An EAD was identified as abnormal depolarization during phase 2 or phase 3 and caused by an increase in the frequency of abortive APs before normal repolarization was completed.

All electrophysiology results are expressed as mean ± standard error of the mean. An unpaired two-tailed Student’s t-test or a one-way ANOVA were used as appropriate. The post-test used was Bonferroni’s HSD (highest significant difference) post hoc test. Statistical analysis was performed using SigmaPlot v14 (Systat Software Inc., CA, USA). A *p*-value inferior to 0.05 was considered statistically significant.

### Cardiomyocyte Ca^2+^ transient measurement

An aliquot of cardiomyocytes suspension (approx. 500 cells) was loaded in standard myocyte Tyrode solution (contained in mM: NaCl 145, KCl 4, CaCl_2_ 1.8, MgCl_2_ 1, glucose 11.1 and HEPES 10, pH 7.4 with NaOH) with 7 µM of Cal520 formulated with 0.05% F-127 Pluronic, kept in the dark at room temperature for 25 min and then transferred to a perfusion chamber (FHC Inc., Bowdoin, ME, USA) mounted on the stage of an inverted Olympus IX73 microscope. Excess of Cal520 was washed out for 5 min with continuous perfusion from a 1–2 mL/min gravity-fed system of standard myocyte Tyrode solution heated to approx. 35 °C using a line heater (Warner Instruments LLC, CT, USA). The cardiomyocytes were field stimulated with a supra-threshold voltage at a 1 Hz pacing frequency, with a bipolar pulse of 3 ms duration, using a pair of platinum wires placed on opposite sides of the chamber connected to a MyoPacer EP stimulator (IonOptix LLC, MA, USA). Starting at 5 V, the amplitude of the stimulating pulse was increased until the cardiomyocytes started generating Ca^2+^ transient cycles, and a value of 1.5-fold the threshold was used throughout an experiment. Cardiomyocytes were then imaged at 20 Hz using an PCO.EDGE sCMOS camera (PCO-TECH Inc., DE, USA) and digitized images were displayed within the MetaMorph acquisition software (Molecular Devices, CA, USA). Responsive cardiomyocytes were selected as regions of interest (ROI) and pixel intensity data was collected from user-defined ROIs.

While perfusing the cells with vehicle control solution (standard myocyte Tyrode solution + 0.1% DMSO), Ca^2+^ transients were acquired for 10 s at 20 Hz frame rate. Drugs were first added to the cells without image acquisition for 5 min; subsequently, while still perfusing the drugs and stimulating the cells, 10 s of imaging data were acquired at 20 Hz rate. For each baseline and drug compound application, all the responsive cells were selected as individual by ROIs. Additional offline analysis, using a validated custom written MatLab program, was carried out to quantify the peak amplitude of the Ca^2+^signal. Data for each experimental condition were the mean of 10 Ca^2+^ peak amplitudes. Results are expressed as mean % change (± standard error) in fluorescent intensity relative to the myocyte’s specific baseline control period (ΔF/F_0_). An EAD-like arrhythmic Ca^2+^ event, abbreviated as arrhythmia in the "Results" section, was visually identified as change in the slope of the Ca^2+^ transient that occurred before the next stimulus-induced transient. Presence or absence of EAD-like arrhythmic Ca^2+^ events was determined by examining non-averaged transients for the 10 s application test article concentration. EAD-like arrhythmic Ca^2+^ event was expressed as incidence: number of cells showing events normalized by the total number of cardiomyocytes. Differences were tested for statistical significance using the paired Student’s t-test. A value of *p* < 0.05 was considered significant.

### Cardiomyocyte contractility measurement

Contractility transients were measured as previously described^11,14^. Briefly, cardiomyocytes were placed in a perfusion chamber (FHC Inc., Bowdoin, ME, USA) mounted on the stage of an inverted Motic AE31E microscope (Stellar Scientific, MD, USA) and continuously perfused from a gravity fed system at 2 ml/min with myocyte Tyrode solution (see composition in Cardiomyocyte Ca^2+^ transient measurement) heated to approximately 35 °C using an inline heater (Cell MicroControls, Norfolk, VA, USA). A video-based cell geometry system was used to measure sarcomere dynamics (IonOptix LLC (v7.2.7.138), MA, USA, www.ionoptix.com) ^11,14^. The myocytes were field stimulated at voltage 50% above threshold at a 1 Hz pacing frequency, with a biphasic pulse of 3 ms duration, using a pair of platinum wires placed on opposite sides of the chamber and connected to a MyoPacer EP stimulator (IonOptix LLC, MA, USA). Images were acquired at a rate of 240 Hz using an IonOptix MyoCam-S CCD camera. Digitized images were displayed within the IonWizard acquisition software (IonOptix LLC, MA, USA). Optical intensity data were collected from a user-defined rectangular region of interest placed over the myocyte image. The optical intensity data represent the bright and dark bands corresponding to the Z-bands of the cardiomyocyte. The IonWizard software (v1.2.22) analyzed the periodicity in the optical density along the myocyte detecting the Z-bands by means of a fast Fourier transform algorithm.

The stability of sarcomere shortening transients was assessed by continuous recording for 120 s in Tyrode’s solution, thereby establishing the vehicle control (in 0.1% dimethyl sulfoxide, DMSO). Subsequently, the test article concentration was applied for a 300 s period. When applicable, four ascending concentrations of the test article were used, providing cumulative concentration-effect (C-E) curves. Analysis was performed using the IonWizard software/Transient Analysis Tool A series of polynomials were fitted to the 5 different phases of the monotonic transient^11,14^. For each test condition, the value of sarcomere shortening was calculated from the average of the last 15 contractions and used to quantify test article-induced effects. Treatment effects on sarcomere shortening were expressed relative to the myocyte’s specific baseline control period. Results are expressed as mean ± standard error. Hill curve was fitted to sarcomere shortening C-E data using SigmaPlot v14.0, (Systat Software Inc., CA, USA, www.systatsoftware.com) ^11,14^ and used to determine EC_50_ (i.e., concentration inducing 50% increase in sarcomere shortening). Aftercontraction and contraction failure were also used to quantify article-induced effects^11,14^. An Aftercontraction was visually identified as change in the slope of the contractility transient that occurred before the next stimulus-induced contraction. Contraction failure was also visually identified when the electrical stimulus did not result in a contraction transient. Presence or absence of Aftercontraction and Contraction failure events was determined by examining non-averaged transients for the last 100 s application of a test article concentration. Aftercontraction and Contraction failure were expressed as incidence: number of transients showing events normalized by the total number of 100 transients. Differences were tested for statistical significance using the paired Student’s t-test. A value of *p* < 0.05 was considered significant.

### Trabeculae force of contraction measurement

Contraction/relaxation cycles were measured as previously described^82^. Briefly, atrial and ventricular trabeculae were dissected from the inner endocardial wall of the atria and ventricles of normal donor hearts and donor hearts with atrial fibrillation, and using micro-scissors and tweezers, and transferred to the recording chambers. The ends of each trabecula were tied with a silk thread and mounted in vertical double-jacketed organ baths containing 25 ml of oxygenated (95% O_2_/5% CO_2_ mixture) Tyrode’s solution (contained in mM: NaCl 136, KCl 4, CaCl_2_ 1.8, MgCl_2_ 0.5, dextrose 11.1, HEPES 10, NaHCO_3_ 12 and NaH_2_PO_4_ 0.35, pH 7.4 with NaOH) warmed to approximately 36 °C. One extremity was attached to an isometric force transducer and the other to the base screw of the stimulation holder. Trabeculae were equilibrated for 30 to 60 min, with continuous oxygenation, while applying approximately a 1 gr. tension. During the initial equilibration, Tyrode solution was changed twice every 30 min. To initiate contraction/relaxation cycles, trabeculae were stimulated through a DS8000 digital stimulator (World Precision Instruments, FL, USA) and field platinum electrodes mounted within each double-jacketed organ bath at a pacing rate of 1 Hz with a voltage of 1.5 × threshold of the amplitude of the stimulating pulse and a unipolar pulse width of 2–3 ms. Recordings were performed in continuous mode with sampling at 10 kHz using LabChart Software (ADInstruments Inc., CO, USA).

Baseline stability of contraction-relaxation cycles was assessed by continuous recording for 20 min in Tyrode’s solution establishing our control vehicle (0.1% DMSO) condition. Subsequently, ATX-II was applied for the same 20 min recording period alone or in combination with GS-967. Although data for ATX-II experimental condition alone were expressed as the mean of 30 consecutive contractions acquired before the manifestation of the first Aftercontraction event, data for ATX-II in combination with GS-967 were expressed as the mean of 30 consecutive contractions acquired at the end of the 20 min period. From this analysis, maximum amplitude of contraction (Max. ampl. contraction; gr) and Aftercontraction incidence were determined. An Aftercontraction was visually identified as change in the slope of the contraction/relaxation cycle that occurred before the next stimulus-induced cycle. Results are expressed as mean ± standard error. Treatment effects on Max. ampl. contraction were expressed relative to the trabeculae’s specific baseline control period. Presence or absence of aftercontraction was determined by examining non-averaged contraction/relaxation cycles for the last 100 s application of a test article concentration. Aftercontraction was expressed as incidence: number of cycles showing Aftercontraction normalized by the total number of 100 cycles. Differences in Max. ampl. contraction were tested for statistical significance using the paired Student’s t-test. A value of *p* < 0.05 was considered significant.

### Test articles

The reference drugs selected for this investigation were obtained from Sigma (CA, USA), Tocris Bioscience (MN, USA) and Cayman Chemical (MI, USA). Drugs were initially formulated in DMSO as a 1000 × stock solution. Stock solutions were diluted to the working concentrations in 0.1% DMSO on the day of the experiment.

## Supplementary Information

Supplementary Information 1.

## Data Availability

Data supporting the findings of this manuscript are available from the corresponding author upon reasonable request. A reporting summary for this Article is available as a Supplementary Information file.
