# Supplementary material for: Arrhythmogenic and antiarrhythmic actions of late sustained sodium current in the adult human heart
Source: Sci Rep. 2021 Jun 8;11:12014. doi: 10.1038/s41598-021-91528-1 (PMC8187365; doi:10.1038/s41598-021-91528-1)
Supplement: Supplementary file 1 — Supplementary Information 1. [file 41598_2021_91528_MOESM1_ESM.pdf]

Arrhythmogenic and antiarrhythmic actions of late sustained sodium current in the adult human heart

Anh Tuan Ton[1,2], William Nguyen[1,2], Katrina Sweat[1,2], Yannick Miron[1], Eduardo Hernandez[1], Tiara Wong[1], Valentyna Geft[1], Andrew Macias[1], Ana Espinoza[1], Ky Truong[1], Lana Rasoul[1], Alexa Stafford[1], Tamara Cotta[1], Christina Mai[1], Tim Indersmitten[1], Guy Page[1], Paul E Miller[1], Andre Ghetti[1] & Najah Abi-Gerges[1*]

[1] AnaBios Corporation, San Diego, CA 92109, USA

Running title: Late sustained sodium current and human heart

[*]Materials and Correspondence should be addressed to:
Dr. Najah Abi-Gerges
3030 Bunker Hill St., Suite 312
San Diego, CA 92109 USA
Tel: +1 858-366-8374
Email: Najah.abigerges@anabios.com

[2]These authors contributed equally: Anh Tuan Ton, William Nguyen, Katrina Sweat

Supplementary Table 1 Donor characteristics

Heart #	Donor identifier	Heart	Age	Sex	Ethnicity	BMI	COD	EF (%)
1	191117HHA	N	57	F	Caucasian	32.2	Head trauma	N/A[a]
2	191202HHA	N	42	M	African American	20.7	CVA/ICH/Stroke	55
3	191206HHA	N	46	F	Caucasian	17.7	CVA/ICH/Stroke	80
4	191212HHA	N	51	M	African American	34.6	CVA/ICH/Stroke	65
5	200106HHA	N	27	F	Asian American	30.9	Head trauma	60
6	200211HHA	N	55	F	Caucasian	29.6	CVA/ICH/Stroke	60
7	200307HHA	N	54	M	African American	37.7	CVA/ICH/Stroke	55
8	200417HHA	N	22	F	Caucasian	21.0	Anoxia/AS	50
9	200602HHA	N	60	M	African American	22.7	CVA/ICH/Stroke	74
10	200619HHB	N	58	F	Caucasian	41.8	Anoxia/CVS	61
11	200623HHA	AF	55	M	African American	42.1	Anoxia/CVS	N/A[a]
12	200708HHA	N	54	M	Caucasian	30.6	CVA/ICH/Stroke	60
13	200812HHA	N	49	F	Caucasian	33.3	CVA/ICH/Stroke	N/A[a]
14	200331HHA	N	46	M	Hispanic	30.7	Head trauma	64
15	201203HHA	AF	62	F	Caucasian	20.6	CVA/ICH/Stroke	65
16	210107HHA	AF	65	M	Caucasian	29.0	CVA/ICH/Stroke	N/A[a]

F, Female; M, Male; BMI, Body Mass Index; COD, Cause Of Death; EF, Ejection Fraction; CVA, Cerebrovascular Accident; ICH, Intracranial Hemorrhage; AS, Asphyxiation; AF, Atrial Fibrillation; HH, Human Heart; HHA, the 1st heart received on the day; HHB, the 2nd heart received the same day; [a]Organ procurement organization could not transplant the heart and consequently no echocardiography was performed; N/A, Not available.

Supplementary Figure 1

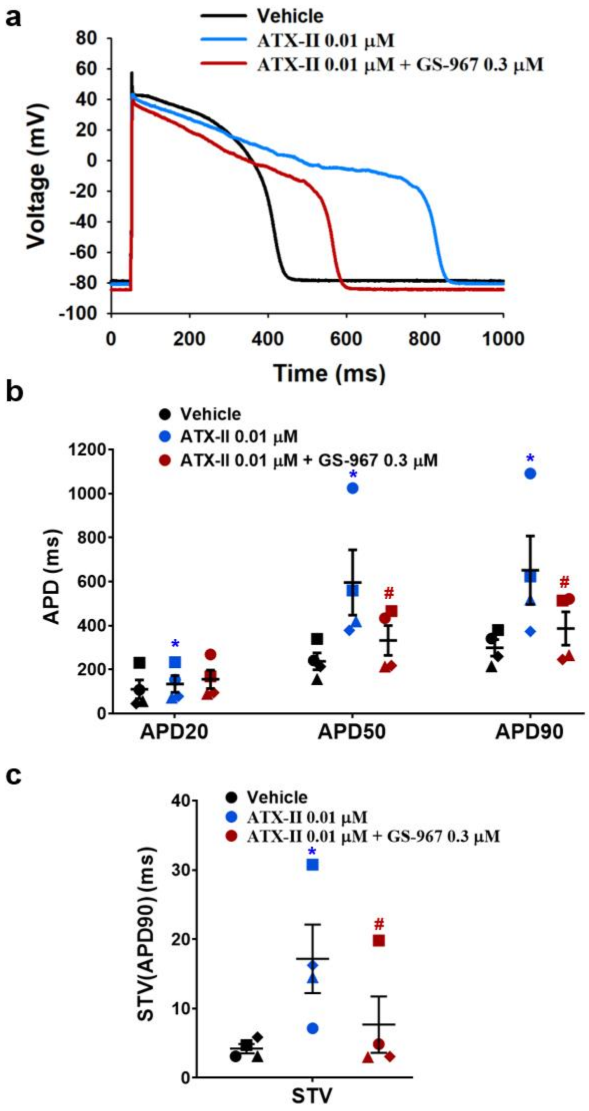

Supplementary Fig. 1 Effects of GS-967 on human ventricular action potential in condition mimicking LQTS3. **(a)** Typical action potentials (APs) recorded from a ventricular cell at a pacing of 1 Hz in the presence of vehicle control and after exposure to 0.01 µM ATX-II alone or in combination with 0.3 µM GS-967. **(b)** and **(c)** show mean % changes in APD (measured at 20% (APD20), 50% (APD50) and 90% (APD90) of repolarization) and STV(APD90), short-term variability of APD90 induced by addition of ATX-II alone or in the presence of GS-967 (n = 4 cells) at 1 Hz. $^{*}p < 0.05$ versus values from vehicle control. $^{\#}p < 0.05$ versus values from ATX-II. Fitmaster analysis software package (HEKA Elektronik, Germany, www.heka.com) and SigmaPlot v14.0 (Systat Software Inc., CA, USA, www.systatsoftware.com) were used to generate the representative AP traces.

Supplementary Figure 2

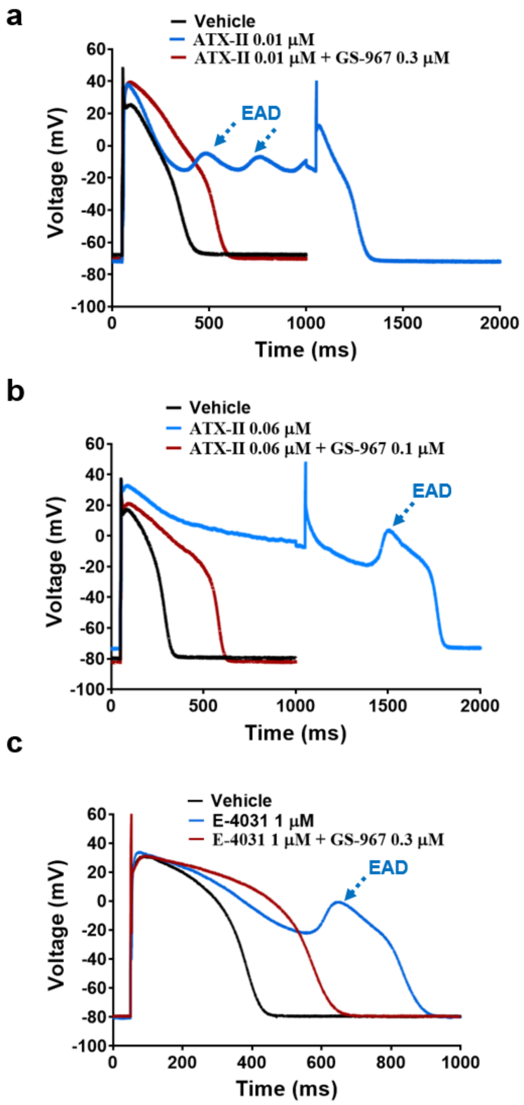

Supplementary Fig. 2 Effects of GS-967 on incidence of early afterdepolarization in conditions mimicking LQTS3 (ATX-II) and LQTS2 (E-4031). **(a)** Typical action potentials (APs) recorded from a ventricular cell at a pacing of 1 Hz in the presence of vehicle control and after exposure to 0.01 µM ATX-II alone or in combination with 0.3 µM GS-967. **(b)** Typical APs recorded from a ventricular cell at a pacing of 1 Hz in the presence of vehicle control and after exposure to 0.06 µM ATX-II alone or in combination with 0.1 µM GS-967. **(c)** Typical APs recorded from a ventricular cell at a pacing of 1 Hz in the presence of vehicle control and after exposure to 1 µM E-4031 alone or in combination with 0.3 µM GS-967. Fitmaster analysis software package (HEKA Elektronik, Germany, www.heka.com) and SigmaPlot v14.0 (Systat Software Inc., CA, USA, www.systatsoftware.com) were used to generate the representative AP traces.

Supplementary Figure 3

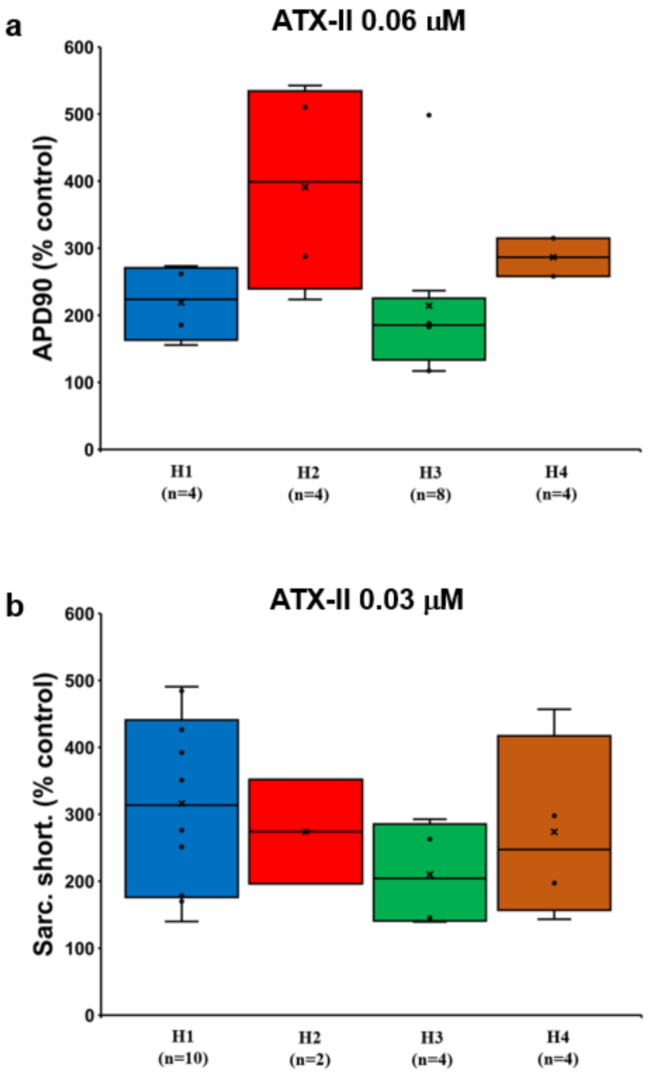

Supplementary Fig. 3 Intra-heart in ATX-II-induced increases in APD90 and sarcomere shortening. **(a)** Intra-heart variability in 0.06 µM ATX-II-induced APD90 (90% of repolarization of the action potential) increases in adult human primary cardiomyocytes from 4 donor hearts. **(b)** Intra-heart variability in 0.03 µM ATX-II-induced increases in sarcomere shortening in adult human primary cardiomyocytes from 4 donor hearts. Each Whisker plot shows the minimum, first quartile, median (horizontal line inside the box), mean (marked by “x”), third quartile and maximum for each donor heart. H: Heart; n: Number of cells per donor heart. GraphPad Prism v7.04 (GraphPad Software, CA, USA) was used to generate the Whisker plots. Statistical analysis was conducted by one-way ANOVA within Excel.

Supplementary Figure 4

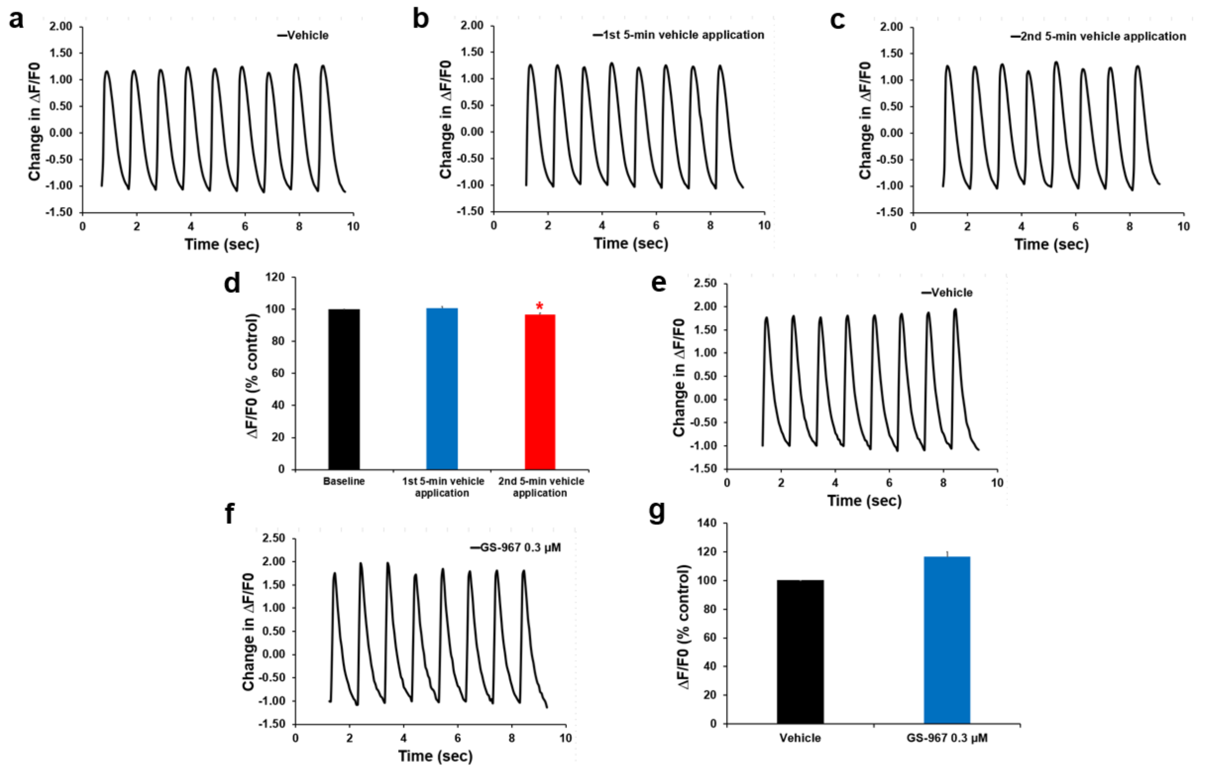

Supplementary Fig. 4 Stability over time (a-c) and effects of GS-967 on ventricular Ca^{2+} transient recordings. **(a-c)** Typical Ca^{2+} transients recorded from a ventricular cell at a pacing of 1 Hz in the presence of vehicle control (**a**) and after exposure to two sequential 5-minute applications of vehicle: 1st (**b**) and 2nd (**c**). **(d)** Mean % change in the amplitude of the Ca^{2+} signal induced by two sequential additions of vehicle at 1 Hz (n= 142 cells). **(e)** and **(f)** Typical Ca^{2+} transients recorded from a ventricular cell at a pacing of 1 Hz in the presence of vehicle control (**e**) and after exposure to 0.3 µM GS-967 (**f**). **(g)** Mean % change in the amplitude of the Ca^{2+} signal induced by addition of 0.3 µM GS-967at 1 Hz (n = 19 cells). $^{*}p < 0.05$ versus values from vehicle control. MetaMorph analysis software (Molecular Devices, CA, USA, www.moleculardevices.com) and a validated custom written MatLab program (The MathWorks Inc., MA, USA, www.mathworks.com) were used to generate the representative Ca^{2+} transients.

Supplementary Figure 5

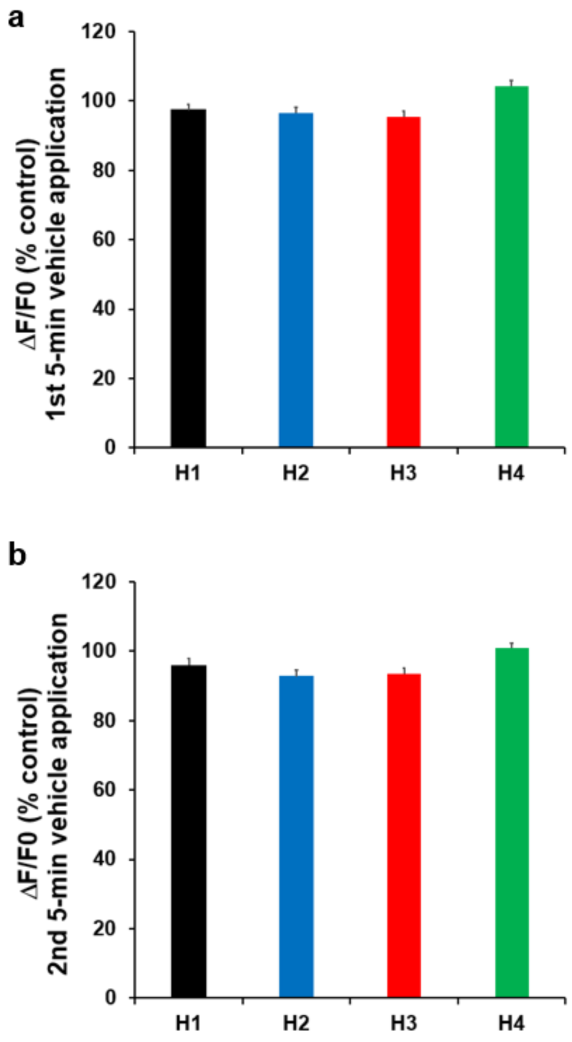

Supplementary Fig. 5 Intra-heart variability in stability over time of ventricular Ca^{2+} recordings. **(a)** and **(b)** Intra-heart variability in vehicle-induced changes in sarcomere shortening of adult human primary cardiomyocytes from 4 donor hearts after exposure to two sequential 5-minute additions of vehicle: 1st (**a**) and 2nd (**b**). H: Heart; n = 31, 30, 22 and 43 cells from H1, H2, H3 and H4, respectively.

Supplementary Figure 6

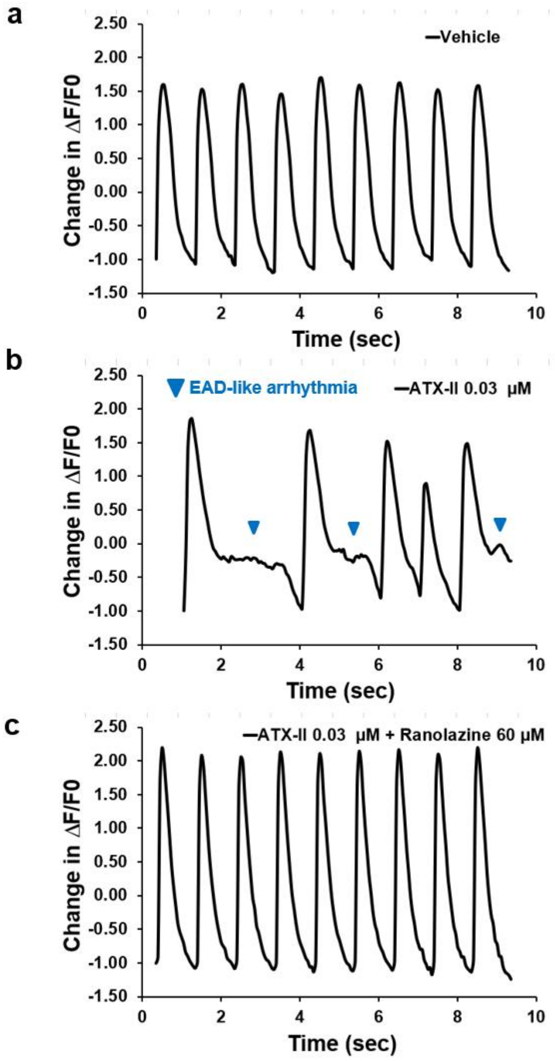

Supplementary Fig. 6 Effect of ranolazine on human ventricular Ca^{2+} transient in condition mimicking LQTS3. **(a-c)** Typical Ca^{2+} transients recorded from a ventricular cell at a pacing of 1 Hz in the presence of vehicle control (**a**) and after exposure to 0.03 µM ATX-II alone (**b**) or in combination with 60 µM ranolazine (**c**). MetaMorph analysis software (Molecular Devices, CA, USA, www.moleculardevices.com) and a validated custom written MatLab program (The MathWorks Inc., MA, USA, www.mathworks.com) were used to generate the representative Ca^{2+} transients.

Supplementary Figure 7

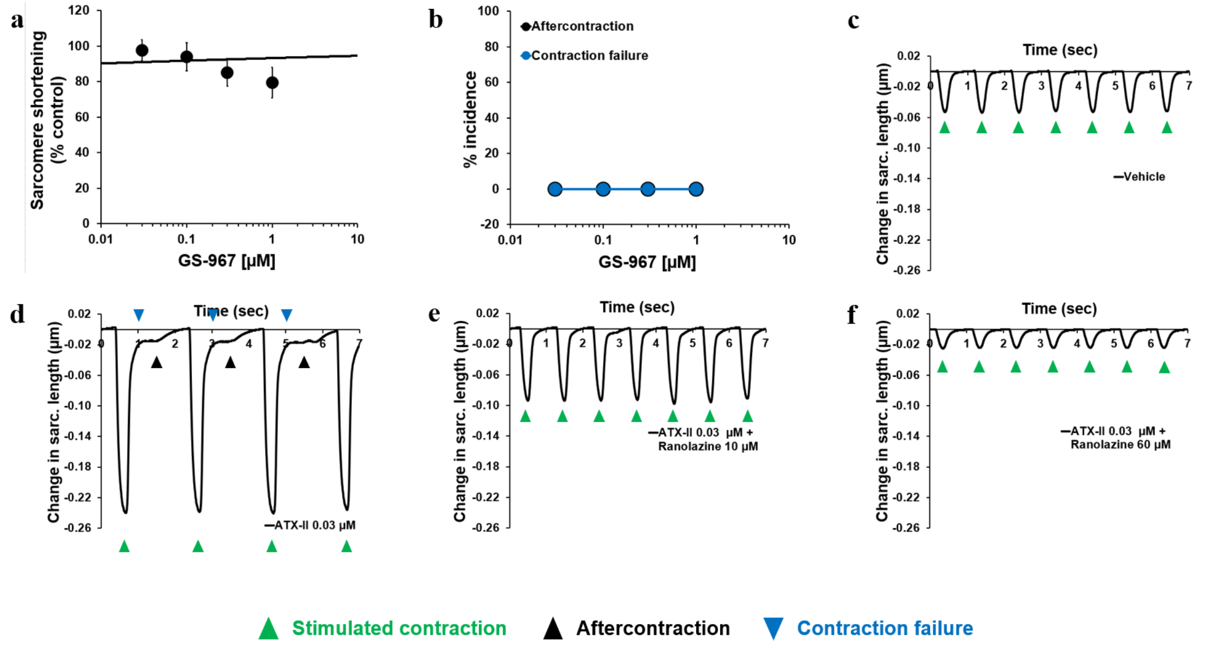

Supplementary Fig. 7 Effects of GS-967 on normal ventricular contractility. **(a)** and **(b)** Mean % change in sarcomere shortening (**a**) and incidence of aftercontraction and contraction failure (**b**) as a function of concentrations tested. Effects of ranolazine on ventricular contractility in condition mimicking LQTS3. (**c**,**f)** Typical contractility transients recorded from a ventricular cell at a pacing of 1 Hz in the presence of vehicle control (**c**) and after exposure to 0.03 µM ATX-II alone (**d**) or in combination with 10 µM (**f**) and 60 µM (**g**) ranolazine. IonWizard software (v1.2.22, IonOptix LLC, MA, USA, www.ionoptix.com) and SigmaPlot v14.0 (Systat Software Inc., CA, USA, www.systatsoftware.com) were used to generate the representative contractility transients and fitted C-E curve, respectively.

Supplementary Figure 8

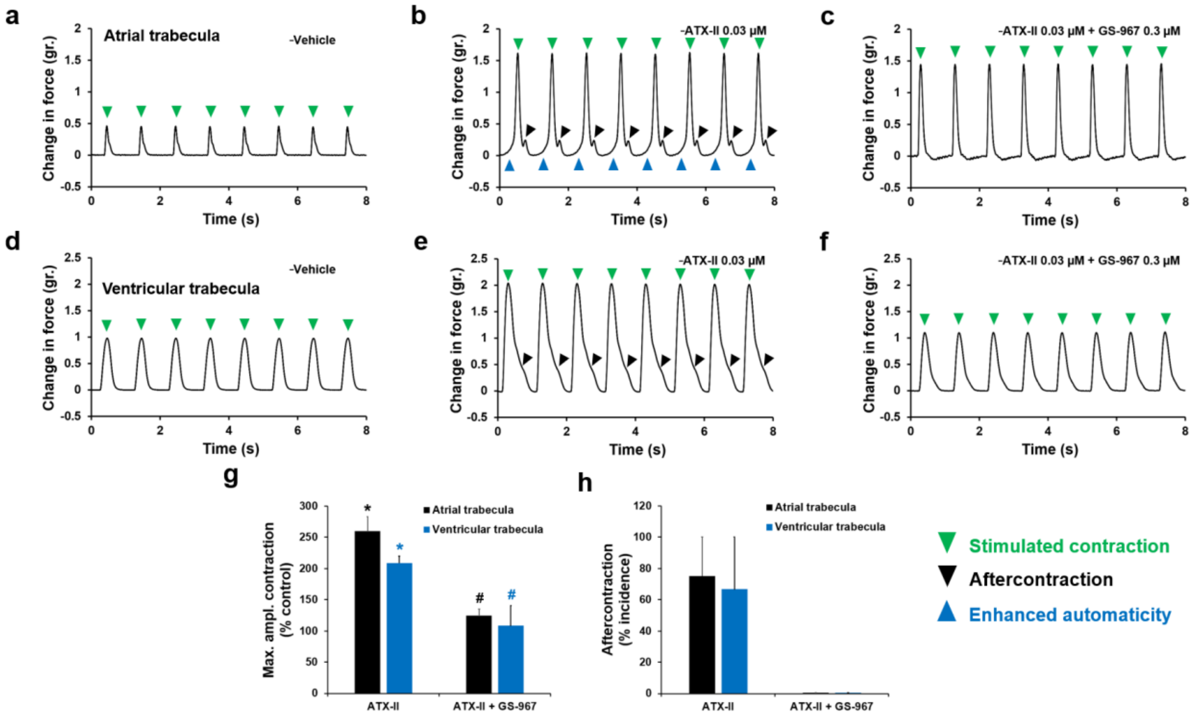

Supplementary Fig. 8 Effects of GS-967 on human atrial and ventricular trabeculae contraction in condition mimicking LQTS3. **(a-c)** Typical contraction/relaxation cycles recorded from an atrial trabecula at a pacing of 1 Hz in the presence of vehicle control (**a**) and after exposure to 0.03 µM ATX-II alone (**b**) or in combination with 0.3 µM GS-967 (**c**). **(d-f)** Typical contraction/relaxation cycles recorded from a ventricular trabecula at a pacing of 1 Hz in the presence of vehicle control (**d**) and after exposure to 0.03 µM ATX-II alone (**e**) or in combination with 0.3 µM GS-967 (**f**). **(g-h)** Mean % control in maximum amplitude of contraction (Max. ampl. Contraction) (**g**) and mean % incidence of aftercontraction (**h**) when atrial and ventricular trabeculae were treated with 0.03 µM ATX-II alone or in combination with 0.3 µM GS-967 (n = 4 and 3 trabeculae, respectively) at 1 Hz. *,*p < 0.05 versus values from vehicle control. #,#p < 0.05 versus values from ATX-II. LabChart Software (v8.1.16, ADInstruments Inc., CO, USA, www.adinstruments.com) was used to generate the representative contractility transients.

Supplementary Figure 9

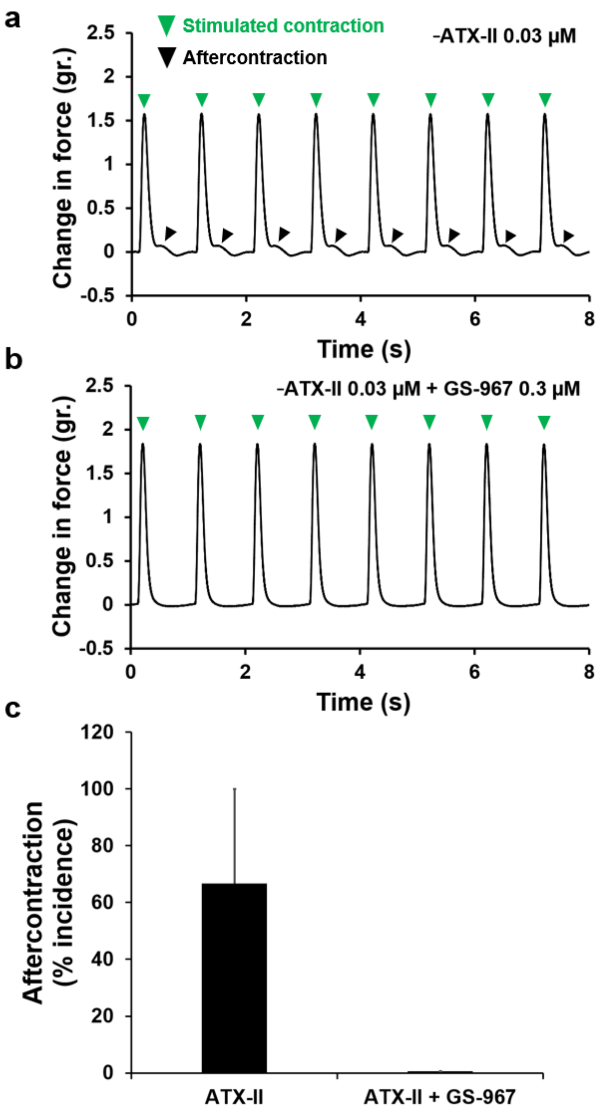

Supplementary Fig. 9 Effects of GS-967 on human atrial trabeculae contraction from donors with atrial fibrillation (AF) in condition mimicking late INa enhancement. **(a**,**b)** show typical contraction/relaxation cycles recorded from an AF atrial trabecula at a pacing of 1 Hz after exposure to 0.03 µM ATX-II alone (**a**) or in combination with 0.3 µM GS-967 (**b**). **(c)** Mean % incidence of aftercontraction when atrial trabeculae were treated with 0.03 µM ATX-II alone or in combination with 0.3 µM GS-967 (n = 3 trabeculae) at 1 Hz. LabChart Software (v8.1.16, ADInstruments Inc., CO, USA, www.adinstruments.com) was used to generate the representative contractility transients.
